# Predictive Modelling of Climate‐Driven Malaria Transmission for Optimal Control Using a Coupled SEIR–SEI and GIS Framework

**DOI:** 10.1002/puh2.70375

**Published:** 2026-09-15

**Authors:** Tafadzwa Chivasa, Mlamuli Dhlamini, Auther Maviza, Wilfred Njabulo Nunu

**Affiliations:** ^1^ Department of Environmental Health Faculty of Environmental Science National University of Science and Technology Bulawayo Zimbabwe; ^2^ Department of Environmental Science Faculty of Environmental Science National University of Science and Technology Bulawayo Zimbabwe; ^3^ Department of Environmental Health Services Ministry of Health and Child Care Mberengwa District Hospital Mberengwa Zimbabwe; ^4^ Department of Applied Mathematics Faculty of Applied Science National University of Science and Technology Bulawayo Zimbabwe; ^5^ Global Change Institute Faculty of Science University of Witwatersrand Johannesburg South Africa; ^6^ Department of Environmental Health School of Public Health Faculty of Health Sciences University of Botswana Gaborone Botswana; ^7^ Division of Public Health Faculty of Health Sciences University of the Free State Bloemfontein South Africa

**Keywords:** climate change, malaria, Mberengwa district, projection, SEIRS–GIS Model

## Abstract

Climate change is a potent intensifier of vector‐borne disease dynamics; however, its impact on malaria in pre‐elimination settings remains poorly understood. A climate‐sensitive transmission model integrated with 11 years of clinical surveillance and facility‐level spatial risk data was applied to assess malaria elimination prospects in Zimbabwe's Mberengwa District. Under mid‐century warming in a high‐emission scenario, the peak infectious prevalence is projected to increase by more than 4‐fold, and the duration of high‐intensity transmission will increase from 213 to 250 days per year. Decadal seasonal oscillations in case counts were reproduced by the model, whereas recent overestimation reflected intensified elimination activities that suppressed transmission below climate‐driven expectations. Sensitivity analysis identified temperature‐dependent mosquito mortality as the dominant source of uncertainty, with insecticide‐based indoor spraying being the most influential, modifiable intervention. Simulations indicate that high‐coverage spraying alone can achieve elimination, and a fully integrated strategy, including bed nets, spraying and gametocyte‐targeting therapy, can reduce transmission by 85%. Spatial mapping of 37 health facility catchments highlighted five high‐priority hotspots concentrated in the southern and south‐eastern health facilities, where climate exposure and the current burden of disease converge. These results provide a locally informed, climate‐intelligent framework to support malaria elimination in the context of accelerating climate change.

## Plain English Summary

Malaria kills hundreds of thousands of people annually, mostly in Africa. In Mberengwa, a rural district in Zimbabwe, malaria cases have been reduced to very low levels; however, these gains are fragile. Climate change is making conditions more favourable for mosquitoes that spread dengue. A computer simulation using daily temperature records from 2014 to 2023 was created to study how malaria spreads in the district. The simulation accounts for how temperature affects mosquito biting, lifespan, and the time required for the malaria parasite to develop within mosquitoes. It was checked against 11 years of hospital records and successfully reproduced the seasonal patterns of malaria. The results showed that if greenhouse gas emissions remain high, the peak number of infectious people could rise by more than four times by mid‐century, and the period of intense transmission could last an extra 5 weeks each year. Despite this, malaria elimination, where the parasite can no longer maintain itself locally, is still achievable if indoor residual spraying reaches 80% of households. Five health facility areas in the south and west of the district were identified as facing the highest combined risk from the current malaria burden and future climate change, providing health programme managers with a clear guide for prioritising interventions.

## Graphical abstract

A climate‐sensitive SEIR–SEI model coupled with GIS mapping across Zimbabwe's Mberengwa District projected a 4‐fold rise in malaria transmission under mid‐century warming. However, it showed that indoor residual spraying at 80% coverage can still achieve elimination, prioritising five high‐risk catchments for climate‐resilient intervention.

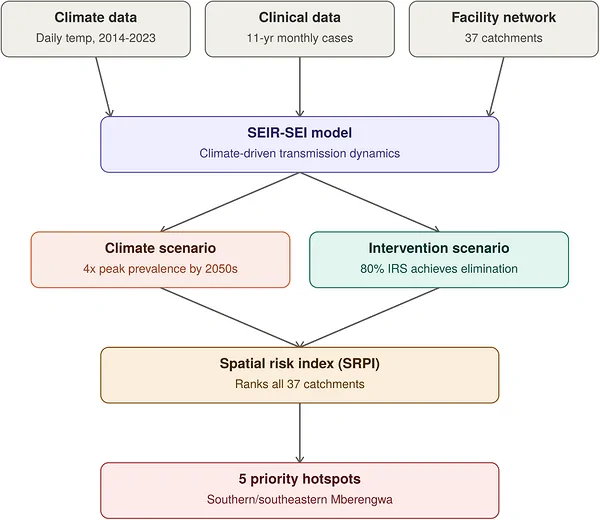

## 1 Introduction

Malaria remains one of the most persistent infectious disease threats globally, responsible for an estimated 249 million clinical cases and 608,000 deaths in 2022, of which 95% occurred in the World Health Organization (WHO) African Region [1]. Despite a 40% reduction in incidence and a 60% reduction in mortality across sub‐Saharan Africa between 2000 and 2022, progress has stalled since 2015, and the absolute number of cases has continued to rise alongside the population growth [2]. Climate change has emerged as a structural threat to these hard‐won gains, operating through direct, non‐linear effects on vector and parasite biology, which modulate the spatial extent, seasonal duration and intensity of *Plasmodium falciparum* transmission [3, 4].

Temperature exerts mechanistic control over every biologically rate‐limiting step in the malaria transmission cycle, including the mosquito biting rate, vector emergence, adult lifespan and extrinsic incubation period of the parasite within the vector. These relationships are fundamentally non‐linear. Laboratory studies of *Anopheles gambiae* and *Anopheles arabiensis* characterised using Brière thermal performance curves (TPCs) show that transmission potential peaks at approximately 25°C and declines sharply at temperatures above 28°C, substantially revising earlier linear estimates that had placed the optimum temperature at 31°C [5]. In semi‐arid environments, where mean temperatures currently occupy the ascending limb of this thermal response curve, marginal warming is predicted to produce a disproportionate amplification of vectorial capacity and transmission intensity, rather than merely extending the geographic range of transmission [6]. Under high‐emission scenarios, climate projections for Africa indicate that an additional 75.9 million people could fall within the zone of endemic transmission in Eastern and Southern Africa by 2080 [7]. A recent study integrating 25 years of African epidemiological and climate data estimated that climate change could generate 123 million additional malaria cases and 532,000 additional deaths on the continent between 2024 and 2050 under current control levels [8].

Zimbabwe exemplifies the vulnerability of pre‐elimination settings in southern Africa to climate‐sensitive transmissions. The country has reduced malaria incidence from 153 cases per 1000 population in 2004 to 9 per 1000 in 2022, with 30 of the 62 districts achieving pre‐elimination status by the end of 2022 [9]. Mberengwa District in Midlands Province reached this threshold in 2018, sustaining an annual parasite index (API) below five cases per 1000 population across its 37 health facility catchments [10]. The district's semi‐arid climate creates conditions in which modest warming is expected to amplify rather than attenuate transmission. Residual transmission persists in spatially isolated foci, and surveillance data document year‐to‐year fluctuations linked to El Niño‐associated temperature anomalies and rainfall variability (Ministry of Health and Child Care [11]). A scoping review of climate‐malaria research in Zimbabwe identified a critical methodological gap, where mechanistic models coupling TPCs to district‐level transmission dynamics are largely absent from the Zimbabwean literature [12]. This limits the capacity to forecast transmission under projected warming and identify the minimum intervention coverage thresholds required for sustained elimination.

Existing quantitative studies on malaria transmission in Zimbabwe and Southern Africa have relied primarily on statistical and spatiotemporal approaches that characterise historical associations between climate variables and case incidence but do not capture the non‐linear physiological mechanisms governing transmission intensity [13, 14]. Statistical regression models applied at district resolution lack the mechanistic structure needed to project transmission under novel climate forcing outside the historical range or to simulate the effect of intervention coverage on transmission potential under future warming conditions. Compartmental ordinary differential equation (ODE) frameworks that explicitly couple TPC forcing to susceptible‐exposed‐infectious‐recovered humans and susceptible‐exposed‐infectious vector dynamics provide the necessary mechanistic resolution but have not previously been developed and validated for a low‐endemic pre‐elimination district in Zimbabwe [5, 15]. Although recent advances in physics‐informed neural networks and hybrid AI‐mechanistic models demonstrate high predictive accuracy for climate‐driven disease forecasting in data‐scarce environments, district‐level mechanistic compartmental models continue to be vital, especially when biological interpretability, intervention thresholds and understanding transmission pathways are important alongside prediction [16, 17].

This study addresses these gaps by developing and validating a climate‐sensitive SEIR–SEI mechanistic transmission model for the Mberengwa District, integrated with geographic risk mapping across all 37 health facility catchment areas in the district. The specific objectives were as follows: (i) to characterise baseline transmission dynamics using TPCs calibrated to ERA5 reanalysis temperature data; (ii) to validate the model against 120 months of confirmed malaria surveillance data from 2014 to 2024; (iii) to identify the structural drivers of transmission potential and disease burden through local and global sensitivity analyses; (iv) to project transmission under SSP2‐4.5 and SSP3‐7.0 warming scenarios from 2021 to 2060; and (v) to identify the minimum integrated intervention coverage required for elimination feasibility under mid‐century warming.

This study makes three key contributions to the evidence base for climate‐adaptive malaria elimination planning in Zimbabwe. First, it presents the first mechanistic, temperature‐driven transmission model built and validated for a low‐endemic, pre‐elimination district in the country, filling a gap identified in the national literature. Second, it moves beyond describing future risk to quantifying action: it identifies the specific combination and coverage level of interventions at which malaria elimination remains biologically achievable under the most demanding mid‐century warming scenario. Third, it translates the modelling outputs into a Spatial Risk Prioritisation Index (SRPI) that ranks all 37 health facility catchments by composite transmission risk, providing programme managers with a practical, data‐driven tool for directing limited resources to where they are most needed.

## 2 Methods

### 2.1 Study Area

This study was conducted in the Mberengwa District, Zimbabwe, a semi‐arid region in the Midlands Province characterised by low annual rainfall (500–700 mm) and high temperatures. The district covers approximately 6047 km^2^ across 37 administrative wards serviced by 37 health facilities [10]. Mean monthly temperatures ranged from approximately 15°C during the cool dry season (June–July) to 27°C during the warm wet season (November–March), based on ERA5 reanalysis data from 2003 to 2023. The district has a resident population of approximately 220,000 [18]. Mberengwa District transitioned to the malaria pre‐elimination phase in 2018 after achieving an API of less than five cases per 1000 population [19]. The primary malaria vector is *A. arabiensis*, which dominates indoor and outdoor transmission across semi‐arid lowland environments in southern Africa [10]. The spatial heterogeneity in transmission risk documented in the preceding spatial analysis motivated the integration of GIS mapping with the mechanistic transmission model developed in this study [20].

### 2.2 Data Sources

Monthly mean near‐surface air temperature data for the Mberengwa District were obtained from the European Centre for Medium‐Range Weather Forecasts Reanalysis fifth Generation (ECMWF‐ERA5) for the period of January 2014 to December 2023. ERA5 provides bias‐corrected, spatially gridded reanalysis data at a 0.25° × 0.25° spatial resolution and has been validated for use in climate‐sensitive disease modelling across sub‐Saharan Africa [21]. Temperature values were extracted at the Mberengwa District centroid (19.5° S, 29.8° E) and aggregated into monthly mean values. No other climate variables were incorporated into the mechanistic model for the reasons stated in Section 2.3.

Monthly confirmed malaria case counts for Mberengwa District were obtained from the Ministry of Health and Child Care Zimbabwe District Health Information System Software (DHIS2) for the period January 2014 to December 2023, yielding 120 monthly observations over 10 years. All confirmed malaria diagnoses (RDT‐positive and slide‐positive), reported through the 37 health facility catchment areas, were included in the study. Data for 2020 were retained in the dataset, but the year was excluded from the analysis because of COVID‐19‐related health service disruptions that altered transmission dynamics [19]. Although the published protocol specified a 2003–2023 data period, pre‐2014 case records exhibited substantial incompleteness and were excluded to preserve data quality and integrity [19].

The year 2020 was excluded from model calibration and validation because COVID‐19‐related restrictions disrupted healthcare access, health‐seeking behaviour and routine health service delivery in Zimbabwe [22]. These disruptions may have affected the comparability of facility‐based monthly malaria case counts with data from other years. To evaluate the sensitivity of the model outputs to this decision, three scenarios were compared: (i) excluding 2020 from the main analysis, (ii) including the raw 2020 observations and (iii) replacing the 2020 observations with linearly interpolated values between the corresponding months of 2019 and 2021. Model performance decreased when the raw 2020 data were included (Pearson's *r* = 0.370; NSE = 0.043) compared to when they were excluded (*r* = 0.544; NSE = 0.212). This suggests that the 2020 observations were less aligned with the model. The scaling factor *k* changed only slightly across scenarios (4.84–5.01), whereas *R*_0_ estimates and SSP3‐7.0 projections remained the same because the thermal forcing and transmission parameters did not depend on *k* (Table S1). Thus, excluding the 2020 improved model enhanced the performance without significantly changing the projected outcomes.

### 2.3 Empirical Basis for Model Development

The SEIR–SEI mechanistic model and the SRPI are grounded in a coordinated chain of evidence from three preceding studies. This multiscale approach ensures that every design choice is traceable to locally generated peer‐reviewed evidence. The specific findings from each study and their direct contributions to the individual model components are summarised in Table 1. First, a national scoping review systematically mapped the impact of climate change on malaria transmission and management in Zimbabwe [10]. This review identified a complete absence of localised mechanistic frameworks in the national literature, confirmed temperature as the dominant thermodynamic constraint on vector and parasite dynamics and provided a biological rationale for the Brière TPC structure adopted in the model. Second, a retrospective case‐based surveillance analysis in the Mberengwa District provided the operational parameter estimates required for a realistic simulation [19]. Most critically, it uncovers an ownership‐use paradox. Despite 95% nominal household LLIN ownership, only 7.7% of confirmed cases demonstrated actual protective use, which directly informed the intervention coverage scenarios evaluated in this model. This further confirmed the district's pre‐elimination quasi‐equilibrium status and identified the April 2020 COVID‐19 disruption breakpoint, which necessitated the exclusion of that year from model validation.

**TABLE 1:** Empirical evidence synthesis guiding the development of the climate‐sensitive SEIR–SEI model.

Study	Design and data	Key finding	Contribution to model
**Chivasa et al**. [12] *Health Services Insights* Scoping review; Zimbabwe; 2000–2024	PRISMA‐ScR scoping review; 22 peer‐reviewed and grey literature sources; PubMed, Web of Science, EBSCO, Cochrane Library, Science Direct	Temperature identified as the dominant biological driver in 19/22 studies; optimal transmission at 25–30°C; warmer conditions shorten the sporogonic period and accelerate vector breeding; vector survival follows a dome‐shaped thermal relationship	Provides the biological rationale for all three Brière thermal performance curves, a(T), πV(T), and μV(T). Further justifies the dome‐shaped functional form of μV(T) and the explicit $E_{V}$ compartment representing the temperature‐dependent extrinsic incubation period
		Plasmodium falciparum confirmed dominant; asymptomatic carriage documented in climate‐favourable areas	Justifies partial immunity parameter (ω = 0.10) in $R_{H}$ and the need for an effective detection fraction (k) linking modelled prevalence to clinical surveillance counts
		No prior study applied mathematical modelling or thermal performance curve frameworks to the climate‐malaria nexus in Zimbabwe	Establishes this SEIR–SEI ODE model as the first mechanistic climate‐driven framework for malaria in Zimbabwe, directly addressing the identified methodological gap
		Climate change driving geographic redistribution of malaria into previously non‐endemic highland areas	Justifies GIS‐based spatial scenario projection via the SRPI to predict transmission expansion under future climate scenarios
**Chivasa et al**. [19] *INQUIRY* Retrospective observational study; Mberengwa; 2019–2024	Individual case‐based surveillance records; DHIS‐2 Tracker, line lists and facility registers triangulated; *N* = 662 confirmed cases; logistic regression and random forest	Significant TPR breakpoint April 2020 (−0.11%/month); pre‐elimination status confirmed (API < 5/1000); seasonal peaks October–March	Pre‐elimination status justifies model initialisation with 10 infectious individuals and the 730‐day spin‐up. The 2020 breakpoint informed exclusion of that year from validation; October–March peaks serve as seasonal benchmarks for Fourier forcing validation
		LLIN ownership 95% vs. self‐reported use 7.7% among confirmed cases.	The 7.7% effective use rate replaces ownership‐based coverage in the intervention efficacy parameter, preventing a 12‐fold overestimation of vector control impact.
		Distance to facility strongest severity predictor (AOR = 0.06, <5 km vs. >10 km; p < 0.001); 10.7% severe cases; 63.7% travel history	Severe case fraction informs $I_{H}$ severity distribution; distance gradient informs SRPI population‐at‐risk weighting; travel history quantifies the importation pathway not captured by the closed population assumption
		Random forest outperformed logistic regression for severity prediction (AUC: 0.76 vs. 0.55; p = 0.003)	Independently justifies the non‐linear ODE approach: superior performance of a non‐linear classifier over linear regression in this population supports a mechanistic rather than statistical modelling framework
**Chivasa et al. (under review)** ^a^ *Manuscript under review* Spatiotemporal analysis; Mberengwa; 2003–2023	Retrospective ecological study; 37 health facility catchments; ERA5, CHIRPS, MODIS NDVI, WorldPop; Global Moran's *I*, LISA, Getis‐Ord Gi*, OLS, SAR, SEM; ArcGIS Pro 2.9 and R 4.5.1	Malaria incidence variability (CV = 176.0%) greatly exceeded that of temperature (CV = 4.0%), rainfall (CV = 29.1%), humidity (CV = 5.7%), and NDVI (CV = 17.1%)	Supports the use of a non‐linear mechanistic framework capable of representing threshold‐dependent amplification of transmission from relatively stable climatic inputs
		Linear spatial regression identified no significant associations between environmental predictors and long‐term incidence change	Motivated the transition from statistical association models to a process‐based compartmental modelling framework capable of representing non‐linear and time‐lagged dynamics
		Annual mixed‐effects modelling identified temperature, rainfall and humidity as significant correlates of absolute incidence	Temperature was selected as the primary forcing variable because of its direct mechanistic influence on vector survival, biting frequency and parasite development
		Global spatial autocorrelation was absent, although local clustering occurred intermittently within south‐eastern facilities	Supported the use of facility‐level spatial units and a patch‐based representation of transmission heterogeneity
		More than one quarter of facilities exhibited temporal trajectories that diverged from district‐level trends	Justified health facility rather than district‐level model parameterisation and validation
		ERA5 provided temporally complete climate records across all facilities throughout the study period	Supported the use of ERA5‐derived temperature forcing for model calibration and validation
		Diagnostic completeness improved substantially following national scale‐up of parasitological confirmation after 2010	Informed restriction of model validation to the post‐2014 period where confirmed‐case surveillance data were more reliable

*Note:* The year 2020 was excluded from model validation owing to COVID‐19‐related disruptions to health service delivery and surveillance reporting. All three studies share institutional ethics approval (NUST/IRB/2024/118).

Abbreviations: AOR, adjusted odds ratio; API, annual parasite index; AUC, area under the curve; CV, coefficient of variation; DHIS‐2, District Health Information System 2; ERA5, ECMWF Reanalysis v5; GIS, geographic information system; LLIN, long‐lasting insecticide‐treated net; NDVI, normalised difference vegetation index; NSE, Nash‐Sutcliffe efficiency; ODE, ordinary differential equation; RMSE, root mean square error; SEIR–SEI, susceptible‐exposed‐infectious‐recovered/susceptible‐exposed‐infectious; SRPI, spatial risk prioritisation index; TPR, test positivity rate.

^a^ Under review; findings described as reported in the submitted manuscript and subject to revision.

Third, a 20‐year longitudinal spatiotemporal analysis covering all 37 health facility catchments in the Mberengwa District confirmed temperature as the main mechanistic climate driver. Linear spatial regression analysis showed no significant links between rainfall, humidity, or NDVI and long‐term changes in malaria incidence, whereas annual mixed‐effects models identified temperature as the strongest factor, along with rainfall and humidity. Consequently, temperature was chosen as the sole forcing variable because of its direct impact on vector survival, biting rate and parasite development, especially as comprehensive facility‐level rainfall data were unavailable for the entire period [19]. This method aligns with recent developments in physics‐informed and AI‐driven climate forecasting, which highlights temperature as the key thermodynamic constraint shaping vector biology and climate‐health interactions in environments with limited data [17, 23]. Spatial autocorrelation analysis further confirmed that transmission occurs within isolated, persistent foci rather than along continuous gradients (Moran's *I* = 0.05–0.13, *p* > 0.05), validating the discrete Voronoi GIS architecture and motivating the facility‐level risk stratification approach of the SRPI.

### 2.4 SEIR–SEI Compartmental Model

#### 2.4.1 Model Structure and Biological Logic

**FIGURE 1:**
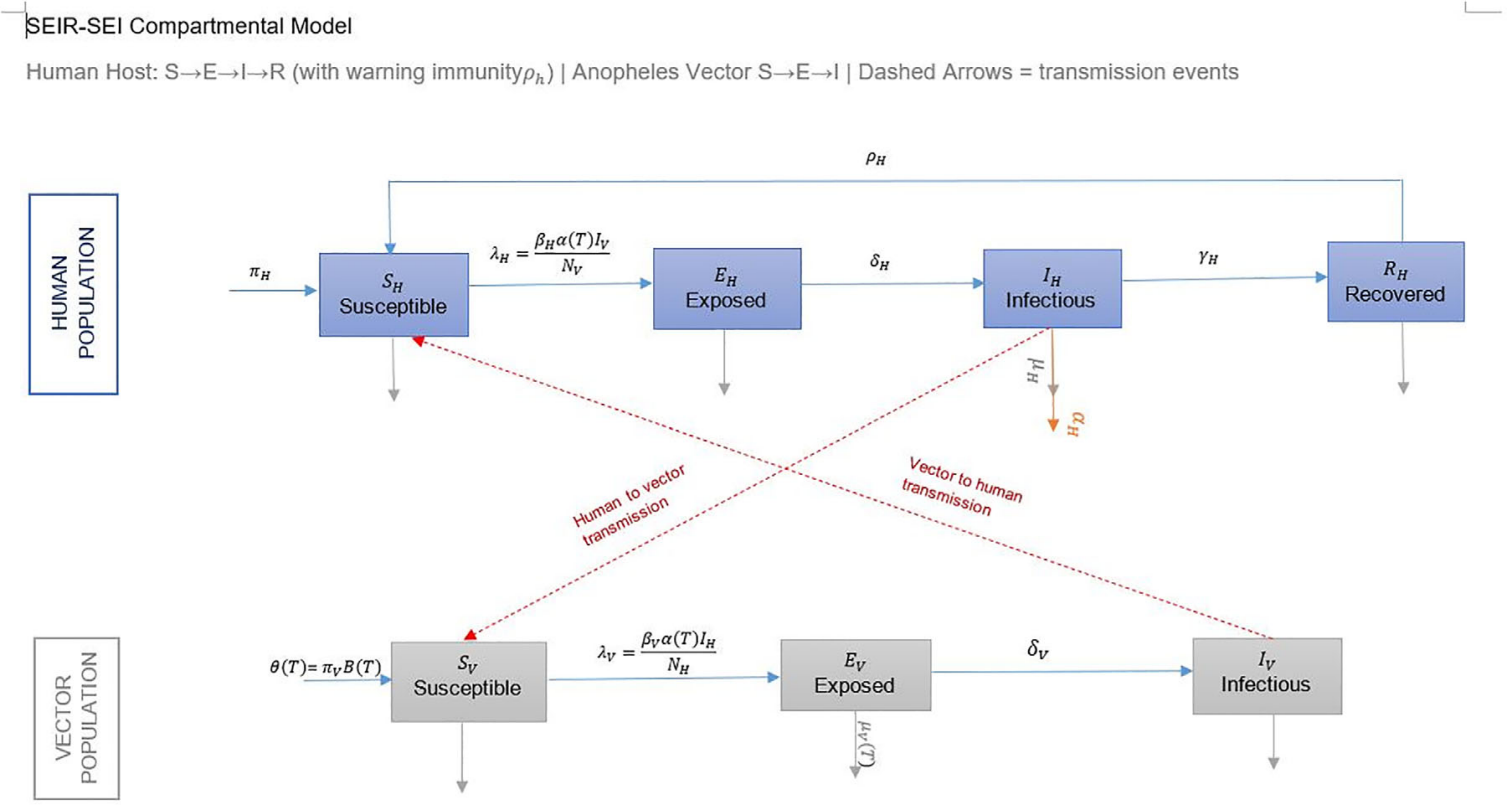
SEIR–SEI model compartments and temperature‐dependent flow.

The model is a deterministic, climate‐sensitive, compartmental ODE system with seven state variables (Figure 1). The model was formulated as three interacting biological sub‐systems, with temperature acting as a key modulator of the underlying processes. The total human population ($N_{H}=220\;000$) is stratified into susceptible ($S_{H}$ *)*, exposed ($E_{H}$), infectious ($I_{H}$) and recovered ($R_{H}$) compartments. Susceptible individuals become exposed at a rate defined by the force of infection ($\lambda_{H}$ *)*, given by the product of the temperature‐dependent biting rate a(T), the vector‐to‐human transmission probability *b* and the proportion of infectious vectors in the population. Exposed individuals progress to the infectious state at rate ($\delta_{H}$ *)*, corresponding to an intrinsic incubation period of approximately 12.5 days and recover at rate ($\gamma_{H}$ *)*, with a mean infectious period of approximately 5 days in a pre‐elimination district that gives both artemisinin‐based therapy (ACTs) and primaquine (PQ) [24]. Recovered individuals retain partial immunity for an average of 142 days and return to the susceptible class at rate $\rho_{H}$ = 0.007 per day. To account for sub‐patent gametocyte carriage, recovered individuals contribute reduced infectiousness, parameterised as a fraction ω=0.10 of that of fully infectious individuals. This contribution $\omega R_{H}$ is incorporated into the human‐to‐vector force of infection and is particularly relevant in Mberengwa's low‐endemic setting, where the recovered compartment remains non‐negligible relative to the infectious class.

The *A. arabiensis* vector population is structured into susceptible ($S_{V}$), exposed ($E_{V}$) and infectious ($I_{V}$) compartments. Vector dynamics are governed by temperature‐dependent recruitment $\pi_{V}\left(T\right)$ and mortality $\mu_{V}\left(T\right)$, ensuring a thermally consistent quasi‐steady‐state population in the absence of infection. The inclusion of the exposed vector compartment enables an explicit representation of the sporogonic period of *P. falciparum*, which is temperature‐dependent and cannot be captured by models that collapse vector dynamics into a single composite transmission term [15]. The SEIR–SEI formulation follows the classical Ross–Macdonald modelling framework and is widely used in mechanistic models of malaria transmission in sub‐Saharan African settings [15, 25]. Figure 1 shows the full schematic of all seven compartments, intercompartment flows, infection forces and temperature‐dependent functions acting on the system.

#### 2.4.2 ODE System

The dynamics of all seven state variables are governed by the following system:

**Human sub‐system**:

(1) $$\frac{dS_{H}}{dt}=\pi_{H}+\rho_{H}R_{H}-\left(\lambda_{H}+\mu_{H}\right)S_{H}$$

(2) $$\frac{dE_{H}}{dt}=\lambda_{H}\;S_{H}-\left(\rho_{H}+\mu_{H}\right)E_{H}$$

(3) $$\frac{dI_{H}}{dt}=\delta_{H}\;E_{H}-\left(\alpha_{H}+\gamma_{H}+\mu_{H}\right)I_{H}$$

(4) $$\frac{dR_{H}}{dt}=\gamma_{H}\;I_{H}-\left(\rho_{H}+\mu_{H}\right)R_{H}$$

**Vector sub‐system**:

(5) $$\frac{dS_{V}}{dt}=\pi_{V}\;\left(T\right)-\left(\lambda_{V}+\mu_{V}\left(T\right)\right)S_{V}$$

(6) $$\frac{dE_{V}}{dt}=\lambda_{V}\;S_{V}-\left(\delta_{V}+\mu_{V}\left(T\right)\right)E_{V}$$

(7) $$\frac{dI_{V}}{dt}=\delta_{V}\;E_{V}-\mu_{V}\left(T\right)I_{V}$$

**Force of infection**:

(8) $$\lambda_{H}=\frac{\beta_{H}\alpha \left(T\right)I_{V}}{N_{V}}\;$$

(9) $$\lambda_{V}=\frac{\beta_{V}\alpha \left(T\right)I_{H}}{N_{H}}\;$$

#### 2.4.3 Thermal Performance Curves

**Mosquito biting rate (** a(T))

The model's sensitivity to climate is driven by three explicitly defined temperature‐dependent functions for the thermal biology of *A. arabiensis*. All TPC parameters were obtained directly from the experimentally characterised curves compiled by Mordecai et al. (2013) [5] and Mordecai et al. (2020) [4, 5].

The mosquito biting rate, a(T), was modelled using a modified exponential function representing a reparameterised Brière‐type thermal response for *A. arabiensis* [5].

(10) $$a\;\left(T\right)=a_{3}\;Te^{-a_{4}T}\;$$

where $a_{3}=\frac{0.04}{25e^{\left(-0.08\times 25\right)}}$ and $\;a_{4}=0.08$. These parameters were calibrated such that $a(25^{\circ }∁)$ was equal to 0.40 bites per vector per day. This biting rate is consistent with empirical estimates for African Anopheles mosquitoes at this temperature [5]. The resulting response captured the increase in biting activity with temperature up to an optimal range, followed by a decline at higher temperatures.

**Adult vector recruitment** $\pi_{V}\left(T\right)$

Adult vector recruitment is represented by a temperature‐dependent function that incorporates the density dependence.

$$\pi_{V}\;\left(T\right)=\pi_{\mathrm{ref}}\;a_{1B}Te^{-a_{2B}T}$$

where $\;a_{1B}=\frac{1}{25e^{\left(-0.06\times 25\right)}}$, $a_{2B}=0.06$ and $\pi_{\mathrm{ref}}=123\;200.$ The scaling parameter $\pi_{\mathrm{ref}}$ is calibrated such that, at the mean annual temperature $\left(22.5^{\circ }∁\right)$, the equilibrium vector population $N_{V}^{*}=\frac{\pi_{V}\left(T\right)}{\mu_{V}\left(T\right)}$ yields a vector‐to‐human ratio consistent with low transmission intensity (API < 5 per 1000 population). This formulation reflects the observed temperature dependence of larval development, with increasing recruitment up to an optimal range and a decline at higher temperatures due to elevated mortality [26].

Adult vector mortality, $\;\mu_{V}\left(T\right)$, is defined as the inverse of temperature‐dependent lifespan [27]:

$$\mu_{V}\;\left(T\right)=\frac{1}{-0.0771T^{2}+3.3292T-11.8239}$$

The quadratic term describes a dome‐shaped relationship between temperature and lifespan, with maximum survival at intermediate temperatures and reduced longevity at thermal extremes. At $\;(22.3^{\circ }∁)$, this yields $\mu_{V}\approx 0.043$ per day, corresponding to a mean lifespan of approximately 23 days. This is consistent with the field estimates of *A. arabiensis* in southern Africa [26]. The model considers a lower bound of 2.5 days on lifespan (and a corresponding upper bound on mortality) to avoid nonbiological values at extreme temperatures.

#### 2.4.4 Seasonal Temperature Model

Daily temperature was modelled using a two‐harmonic Fourier series fitted by ordinary least squares to monthly ERA5 means for 2014–2023.

$$\begin{array}{ccc} T\left(t\right) & = & 22.25+5.52\cos\left(\frac{2\pi t}{12}\right)+1.87\sin\left(\frac{2\pi t}{12}\right)\\ & & -0.67\cos\left(\frac{4\pi t}{12}\right)-1.15\left(\frac{4\pi t}{12}\right) \end{array}$$

where $t=1+\frac{\mathrm{day}}{365}\times 12$. The two‐harmonic formulation captures both the primary annual cycle and the first semi‐annual harmonic, reproducing the observed seasonal asymmetry between the extended cool–dry period (May–September) and the shorter hot–wet season (November–March). The fitted series reproduced the observed temperature range (15.0–27.3°C) with a root‐mean‐square error of 0.18°C.

#### 2.4.5 Basic Reproduction Number

The basic reproduction number $R_{0}\left(t\right)$ was derived analytically using the next‐generation matrix method of van den Driessche and Watmough [28]. This starts by identifying all classes of infection, followed by defining all terms that introduce new infections in matrix F and the remaining terms V. The reproduction number is given by the spectral radius of $FV^{-1}$.

$$F=\left[\begin{array}{cccc} 0 & 0 & 0 & a\left(T\right)\cdot b\\ 0 & 0 & 0 & 0\\ 0 & \frac{a\left(T\right)\cdot c\cdot \pi_{V}\left(T\right)}{\;\mu_{V}\left(T\right)N_{H}^{*}} & 0 & 0\\ 0 & 0 & 0 & 0 \end{array}\right]$$

$$\;V^{-1}=\left[\begin{array}{cccc} \frac{1}{\delta_{H}+\mu_{H}} & 0 & 0 & a\left(T\right)\cdot b\\ \frac{\delta_{H}}{\left[\left(\delta_{H}+\mu_{H}\right)\left(\gamma_{H}+\mu_{H}+\alpha_{H}\right)\right]} & \frac{1}{\left(\gamma_{H}+\mu_{H}+\alpha_{H}\right)} & 0 & 0\\ 0 & 0 & \frac{1}{\left(\delta_{V}+\mu_{V}\left(T\right)\right)} & 0\\ 0 & 0 & \frac{\delta_{V}}{\left[\left(\mu_{V}\left(T\right)\right)\left(\delta_{V}+\mu_{V}\left(T\right)\right)\right]} & \frac{1}{\mu_{V}\left(T\right)} \end{array}\right]$$

Upon computing the spectral radius of $FV^{-1}$, the reproduction number is found to be:

$$\;R_{0}=\sqrt{\frac{a{\left(T\right)}^{2}\cdot b\cdot c\cdot \delta_{H}\cdot \delta_{V}\cdot \pi_{V}\left(T\right)}{\left(\delta_{H}+\mu_{H}\right)\left(\gamma_{H}+\mu_{H}+\alpha_{H}\right)\cdot \mu_{V}{\left(T\right)}^{2}\cdot N_{H}^{*}}}$$

By Theorem 2 of van den Driessche and Watmough [28], $\;R_{0}$ <1 is a necessary and sufficient condition for local asymptotic stability of the disease‐free equilibrium, establishing $R_{0}$ as the threshold parameter for transmission in this system [28].

#### 2.4.6 Initial Conditions

The model was initialised with 10 infectious individuals $I_{H}=10$ in a total human population of 220,000, consistent with Mberengwa's pre‐elimination status (API < 5 per 1000) [10]. The remaining human compartments were set at analytical quasi‐equilibrium values. The initial vector population was defined at the mean annual temperature (22.3°C) as $N_{V0}\approx 3.1\times 10^{6}$ and $I_{V0}=0.005$. A 730‐day spin‐up period was applied prior to each analysis year to allow the system to converge to a seasonally forced endemic quasi‐equilibrium, thereby reducing its sensitivity to the initial conditions.

### 2.5 Model Parameters

All model parameters, including their biological interpretation, values, units and primary literature sources, are presented in Table 2. The vector‐to‐human transmission probability (*b* = 0.10) was selected to reflect intermediate transmission settings, given that reported values range from 0.02 to 0.05 in high‐transmission contexts with substantial acquired immunity to 0.40–0.50 in immunologically naïve populations [15, 25]. This value is consistent with previous mechanistic modelling studies conducted in low‐endemic settings in Southern and Eastern Africa [25].

**TABLE 2:** Model parameters, baseline values, units and sources.

Symbol	Parameter	Value	Units	Source/Justification
$\pi_{H}$	Human recruitment	8.5	Persons per day	Zimbabwean crude birth rate of 28/1000/year was applied to 220,000 [18]
$\mu_{H}$	Human natural mortality	0.000045	Per day	Life expectancy was 61.2 years; 1/(61.2 × 365) [18]
b	Vector‐to‐human transmission probability	0.10	Dimensionless	Low‐endemic partial immunity setting [25]
c	Human‐to‐vector transmission probability	0.35	Dimensionless	[25, 54]
$\;\delta_{H}$	Human latency rate	0.08	Per day	Intrinsic incubation ∼12.5 days [55]
$\gamma_{H}$	Human recovery rate	0.20	Per day	Infectious period ∼5 days under ACTs [56]
$\rho_{H}$	Immunity waning rate	0.007	Per day	Partial immunity half‐life is ∼142 days and is low‐endemic [15]
$\alpha_{H}$	Malaria case fatality rate	0.005	Per day	The malaria CFR is approximately 0.5% [1]
ω	Relative infectiousness of $R_{H}$	0.10	Dimensionless	Sub‐patent gametocytaemia [31]
$\delta_{V}$	Sporogonic rate	0.09	Per day	Extrinsic Incubation PeriodI∼11.1 days at 25°C [5, 57]
$N_{H}$	Total human population	220,000	Persons	[18]

### 2.6 Climate Change Scenarios

Future transmission (2021–2060) was projected under two shared socioeconomic pathway (SSP) scenarios, consistent with the study protocol [10]. For each scenario, a time‐varying temperature offset, ΔT(year) was added to the baseline seasonal forcing:

$$T\;\left(t,\;\mathrm{year}\right)=T_{\mathrm{baseline}}\;\left(t\right)+\Delta T\left(\mathrm{year}\right)$$

The offset was specified as a linear function of time, increasing from its initial value in 2021 to its endpoint of 2060. Under SSP2‐4.5, ΔT increases from +0.5°C to +1.5°C, whereas under SSP3‐7.0, it increases from +0.8°C to +2.8°C. These trajectories represent progressively increasing temperature perturbations, which are consistent with the projected regional warming in southern Africa [29].

### 2.7 Intervention Analysis

Interventions were implemented as direct perturbations of model parameters corresponding to their underlying biological mechanisms. Coverage levels of 30%, 60% and 80% were evaluated for each intervention individually and for all pairwise and combined scenarios (seven combinations across three coverage levels). These coverage levels span the range from current effective coverage to programme targets, as informed by observed ownership‐use disparities [19].

Insecticide‐treated nets (ITNs) were modelled as a reduction in the effective biting rate:

$$a_{\mathrm{eff}}\;\left(T\right)=a\left(T\right)\left(1-e_{\mathrm{itn}}\cdot \mathrm{cov}\right),$$

where $e_{\mathrm{itn}}=0.50$ represents per contact efficacy [30].

Indoor residual spraying (IRS) was incorporated as an additive to increase the vector mortality.

$$\mu_{V,\mathrm{eff}}\;\left(T\right)=\mu_{V}\;\left(T\right)+d_{\mathrm{irs}}\cdot \mathrm{cov},$$

where $d_{\mathrm{irs}}=0.10$ per day denotes the maximum additional mortality attributable to IRS [15].

PQ was modelled using two mechanisms: increased recovery and reduced post‐infection infectiousness, implemented as

$$\;\gamma_{H,\mathrm{eff}}=\gamma_{H}\;\left(1+p_{q,\gamma }\cdot \mathrm{cov}\right),\omega_{\mathrm{eff}}=\omega \left(1-p_{q,\omega }\cdot \mathrm{cov}\right),$$

where $p_{q,\gamma }=0.50$ and $p_{q,\omega }=0.70,$ reflecting its gametocidal effect [31, 32]. Elimination feasibility was defined as achieving a maximum annual mean $R_{o}<1$ under a given intervention scenario, indicating that transmission cannot be sustained in the absence of reintroduction [28].

### 2.8 Model Validation

Model predictions were validated against 120 months of DHIS2‐confirmed case counts (January 2014–December 2023, excluding 2020). Because the model tracks the total infectious prevalence, including asymptomatic and sub‐patent infections, the reported clinical cases represent only a fraction of the true infectious population. This discrepancy was accounted for using a proportional scaling factor, *k*, which quantifies the effective detection fraction that links the modelled prevalence to the reported cases. This factor implicitly captures care‐seeking behaviour, diagnostic testing coverage and reporting completeness. It was estimated over the training period (2014–2019) and held constant for out‐of‐sample validation (2021–2023).

For the validation runs, year‐specific observed monthly temperatures replaced the Fourier baseline and were interpolated to daily values using cubic splines, with each simulation preceded by a 2‐year spin‐up. The model performance was assessed using five metrics according to the established evaluation criteria [33].

To formally establish the identifiability of *k* and quantify its uncertainty, a nonparametric bootstrap procedure was used. A total of 1000 bootstrap samples were drawn with replacement from the 72 monthly observations in the training period, and *k* was re‐estimated for each sample using the proportional regression formula $\;\sum \left(\mathrm{obs}\cdot I_{H}\right)/\sum \left({I_{H}}^{2}\right)$. Because all biological transmission parameters were fixed at values from the literature and were not estimated jointly with *k*, this analysis represents a one‐dimensional identifiability assessment rather than a multi‐parameter correlation problem. The profile likelihood curve for *k* (Figure S1) shows a clearly defined global minimum with no evidence of flat ridges or multiple optima, confirming the structural identifiability. The resulting 95% bootstrap confidence interval [3.850, 6.673] provides uncertainty bounds for the detection fraction.

To highlight the enhanced predictive value of the mechanistic SEIR–SEI framework compared to a simpler statistical method, a seasonal ARIMA model was fitted to the data from 2014 to 2019. This model then generated out‐of‐sample forecasts for 2021–2023. Model order selection was automated using the corrected AIC (AICc) through R's forecast package with *auto.arima()*, considering a seasonal period of 12 months. Forecast accuracy was measured using Pearson's *r*, Nash‐Sutcliffe efficiency and per cent bias on the same 2021–2023 validation data as the SEIR–SEI model, allowing a direct comparison of methodologies on identical held‐out data.

### 2.9 Sensitivity Analysis

#### 2.9.1 Local (One‐at‐a‐Time) Sensitivity

Local (one‐at‐a‐time) sensitivity analysis was conducted by perturbing each of the 14 parameters (Table 3) by ±25% while holding all others fixed. Sensitivity was quantified using the normalised sensitivity index (NSI), defined as the proportional change in the model output relative to the proportional change in the parameter [34]. The evaluated outputs were mean annual $R_{0}$, peak monthly $I_{H}$ and the number of days per year with $R_{0}$>2.

$$\mathrm{NSI}\;=\frac{\Delta \mathrm{Output}/\mathrm{Outpu}t_{\mathrm{baseline}}}{\Delta \mathrm{Parameter}/\mathrm{Paramete}r_{\mathrm{baseline}}}$$

**TABLE 3:** Parameters used in global sensitivity analysis, with baseline values and sampling ranges (*n* = 1000).

Parameter	Description	Baseline	LHS range	Units	Source
**Thermal performance curve parameters**					
$T_{0,a}$	Biting rate lower thermal limit	10.0	8.0–12.0	°C	[5]
$T_{m,a}$	Biting rate upper thermal limit	40.08	37.0–43.0	°C	[5]
$T_{0,\theta }$	Emergence lower thermal limit	10.0	8.0–12.0	°C	[5]
$T_{m,\theta }$	Emergence upper thermal limit	37.73	35.0–40.0	°C	[5]
$q_{1}$	Vector mortality quadratic coefficient 1	0.0771	0.058–0.096	Dimensionless	[58, 59]
$\;q_{2}$	Vector mortality quadratic coefficient 2	3.3292	2.50–4.16	Dimensionless	[58, 59]
**Transmission and host immunity parameters**					
b	Vector‐to‐human transmission probability	0.10	0.05–0.20	Dimensionless	[25]
c	Human‐to‐vector transmission probability	0.35	0.20–0.50	Dimensionless	[25]
$\;\gamma_{H}$	Human recovery rate	0.20	0.10–0.33	Per day	[15, 32]
$\;\rho_{H}$	Immunity waning rate	0.007	0.003–0.014	Per day	[15, 31]
$\;e_{\mathrm{itn}}$	ITN per‐contact efficacy	0.50	0.30–0.70	Dimensionless	[30]
$\;d_{\mathrm{irs}}$	IRS additional vector mortality	0.10	0.05–0.20	Per day	[15]
$\;pq_{\gamma }$	PQ recovery acceleration factor	0.50	0.25–0.75	Dimensionless	[31, 32]
$\;pq_{\omega }$	PQ infectiousness reduction factor	0.70	0.40–0.90	Dimensionless	[31, 32]

Abbreviations: IRS, indoor residual spraying; ITN, insecticide‐treated net; LHS, Latin Hypercube Sampling; PQ, primaquine.

Absolute values of ∣NSI∣>1 indicate amplified sensitivity. All analyses were performed under the SSP3‐7.0 mid‐century scenario (+2.8°C) as a stress‐test condition.

#### 2.9.2 Global Sensitivity Analysis (Latin Hypercube Sampling [LHS]‐PRCC)

Global sensitivity analysis was performed using LHS with *n* = 1000 parameter sets drawn from the joint uncertainty space of 14 parameters. This approach guarantees an even coverage of the full parameter range [35]. Partial rank correlation coefficients (PRCCs) were computed between each parameter and four outputs: mean annual $R_{0}$, peak $I_{H}$, days per year with $R_{0}$>2 and percentage reduction in $R_{0}$ under 80% combined ITN, IRS and PQ coverage. PRCC is a rank‐based measure that accounts for the influence of all other parameters simultaneously and is robust to non‐linear but monotonic relationships [36]. Parameters with absolute PRCCs greater than 0.5 were classified as having a strong influence on the model outputs [35].

Parameter uncertainty was further propagated using 200 LHS samples to generate 50% and 95% credible intervals for seasonal $R_{0}\left(t\right)$ trajectories. Statistical significance was assessed at *α* = 0.05 using a *t*‐test on rank‐transformed correlations with n−2−K degrees of freedom [35]. All analyses were conducted under the SSP3‐7.0 mid‐century scenario with 80% combined intervention coverage.

### 2.10 GIS Spatial Integration

Spatial analysis was conducted in R (version 4.5.1) using the *sf* package for geometric operations, with final maps produced in QGIS (version 3.40.12). Data for the 37 health facility catchments included facility coordinates, annual confirmed malaria cases from 2014 to 2024 (excluding 2020), catchment population estimates and monthly mean temperature. The administrative boundaries for Zimbabwe were sourced from the GADM level‐2 shapefile, from which the Mberengwa District polygon was extracted using an attribute filter. Catchment areas were approximated using Voronoi tessellation of the 37 facility locations because facility‐level catchment boundary shapefiles were not available [37]. Polygons were clipped to the district boundary and assigned to the nearest facility using the spatial nearest feature join. This approach partitions the district into 37 mutually exclusive zones, each encompassing all areas that are geographically closer to its assigned facility than to any other. Voronoi tessellation is widely used in health accessibility analyses when utilisation data are unavailable [38, 39].

Five metrics were computed for each of the catchments. The mean annual malaria incidence (cases per 1000 population per year) was derived from the DHIS2 case counts and population estimates. Baseline mean annual $R_{0}$ and the number of months per year with $R_{0}$(*t*)>1 were computed using facility‐specific monthly mean temperature series. These computations were repeated with warming offsets of +0.8°C (SSP2‐4.5 near‐future) and +1.8°C (SSP3‐7.0 mid‐century) applied to the facility‐specific temperature series, accounting for spatial variation in temperature across the district. The number of months per year with $R_{0}$(*t*)>1 under SSP3–7.0 mid‐century conditions was used as the climate exposure metric in the prioritisation index.

A SRPI was computed for each catchment as the arithmetic mean of three min–max normalised indicators. The first indicator was the mean annual malaria incidence, which represents the current burden of malaria. The second was projected months per year with $R_{0}$(*t*)>1 under SSP3‐7.0, representing climate exposure, and the third was mean annual catchment population, representing the population at risk. The SRPI was computed as follows:

$$\;\mathrm{SRP}I_{i}=\frac{{\tilde{I}}_{i}+{\tilde{R}}_{0,i}+{\tilde{P}}_{i}}{3}$$

where the tildes denote min–max‐normalised values. Facilities were assigned to four intervention priority tiers according to their SRPI scores: Priority 1 (SRPI ≥ 0.75), Priority 2 (0.50–0.74), Priority 3 (0.25–0.49) and Priority 4 (<0.25). Equal weighting across the three indicators was applied because of the lack of an empirical basis for differential weighting in the study setting.

### 2.11 Ethical Considerations

This study received ethical approval from the National University of Science and Technology (NUST) Institutional Review Board, and permission to access malaria surveillance data was granted by the Ministry of Health and Child Care, Zimbabwe. This study utilised anonymised, aggregated secondary data from the DHIS2 surveillance system and publicly available ERA5 climate reanalysis data; no primary data collection involving human participants was undertaken. As all data were de‐identified prior to extraction, individual informed consent was not required for this study. Data were stored securely on password‐protected devices accessible only to the research team and will be retained for 5 years post‐publication before permanent deletion.

## 3 Results

### 3.1 Seasonal Thermal Forcing and Baseline Dynamics

**FIGURE 2:**
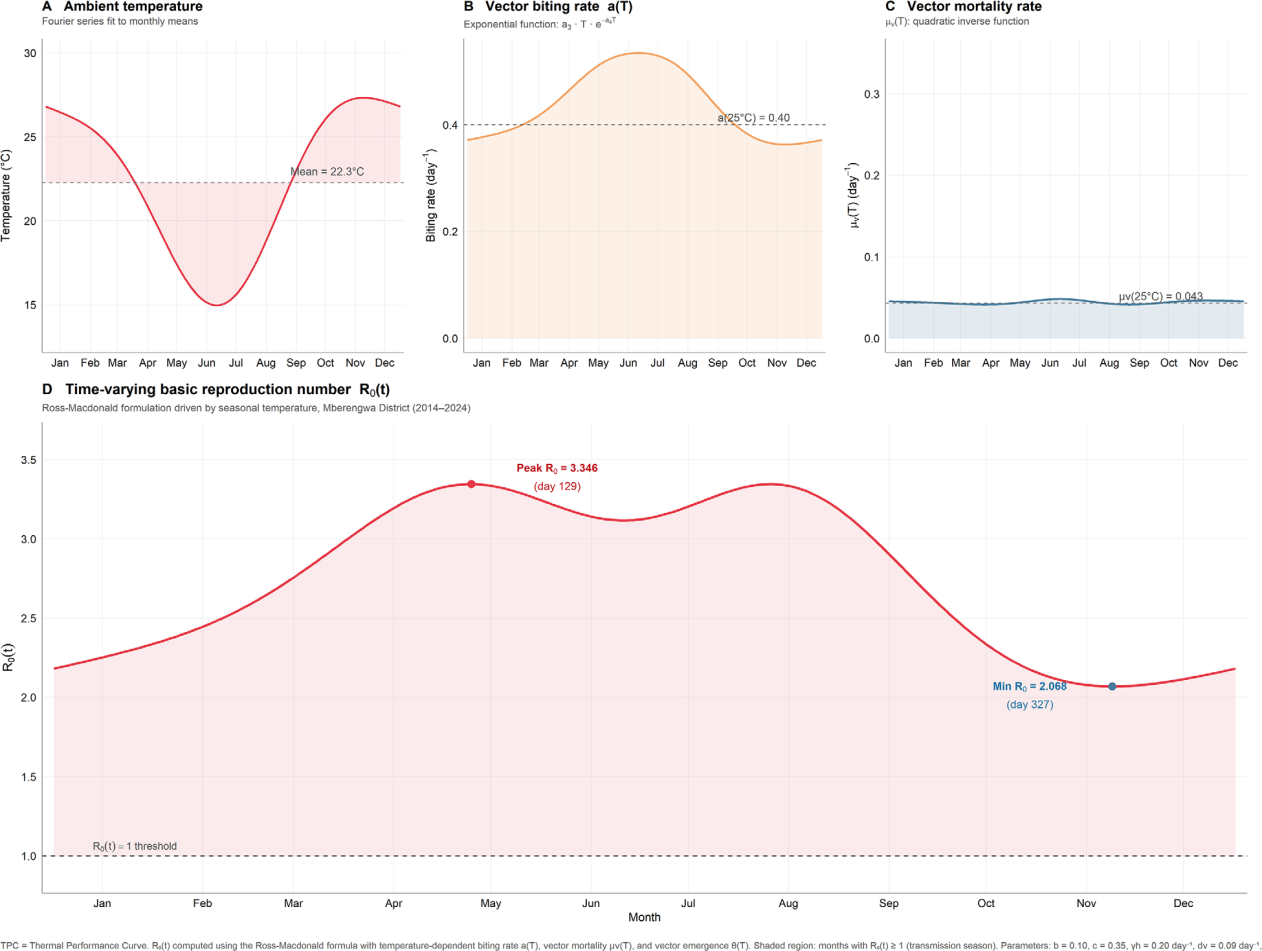
Seasonal temperature, vector biting and mortality, and $R_{0}\left(t\right)$ with seasonal lag. (A‐Ambient Temperature; B‐Vector Biting Rate; C‐Vector Mortality Rate; D‐Time Varying Basic Reproduction Number)

The Fourier series fit characterised the seasonal temperature profile of the Mberengwa District with a root mean square error (RMSE) of 0.18°C. The mean annual temperature of the district was 22.3°C, with monthly means ranging from approximately 15°C in June–July to 27°C in October–November (Figure 2A). The temperature‐dependent vector‐biting rate *a*(*T*) peaked at 0.40 bites vector/day during the warm season (October–April) and reached its annual minimum during the cool season (June–August) (Figure 2B). The vector mortality rate $\mu_{v}\left(T\right)$ ranged from 0.043 day^−1^ at 25°C to 0.05 day^−1^ during the cool season (Figure 2C).

The time‐varying basic reproduction number $R_{0}\left(t\right)$ remained above 1.0 throughout the entire 365‐day cycle, ranging from a minimum of 2.068 on Day 327 (∼November) to a peak of 3.346 on Day 129 (∼May) (Figure 2D). The infectious human population $I_{H}\left(t\right)$ ranged from 1355 to 3179 individuals over the annual cycle. The peak in $I_{H}\left(t\right)$ occurred on Day 215 (∼August), 86 days after the peak in $R_{0}\left(t\right)$ on Day 129 (∼May) (Figure 2D and Figure S2).

### 3.2 Model Validation Against Historical Surveillance (2014–2024)

**FIGURE 3:**
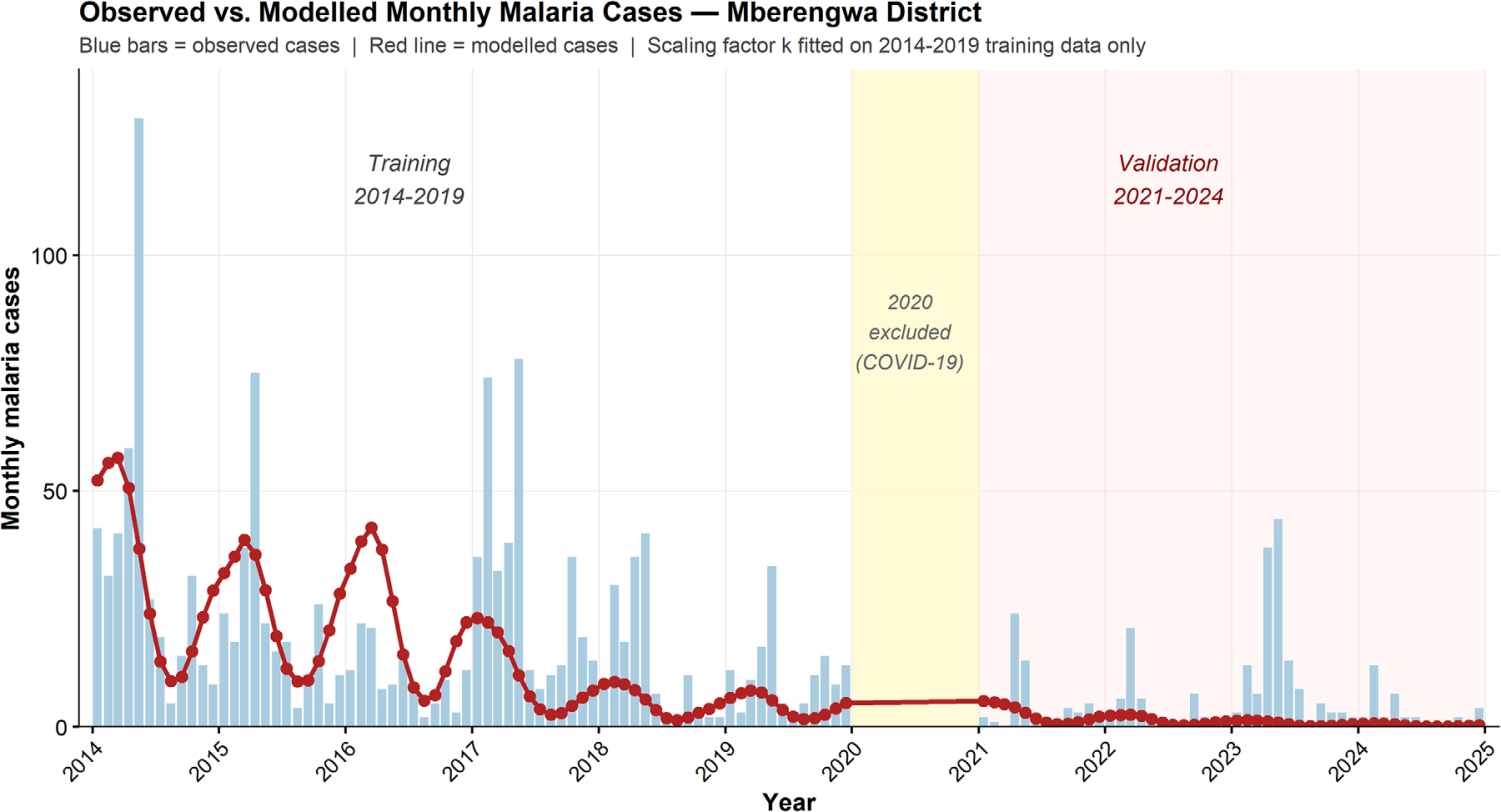
Observed and modelled monthly malaria cases (2014–2024).

**FIGURE 4:**
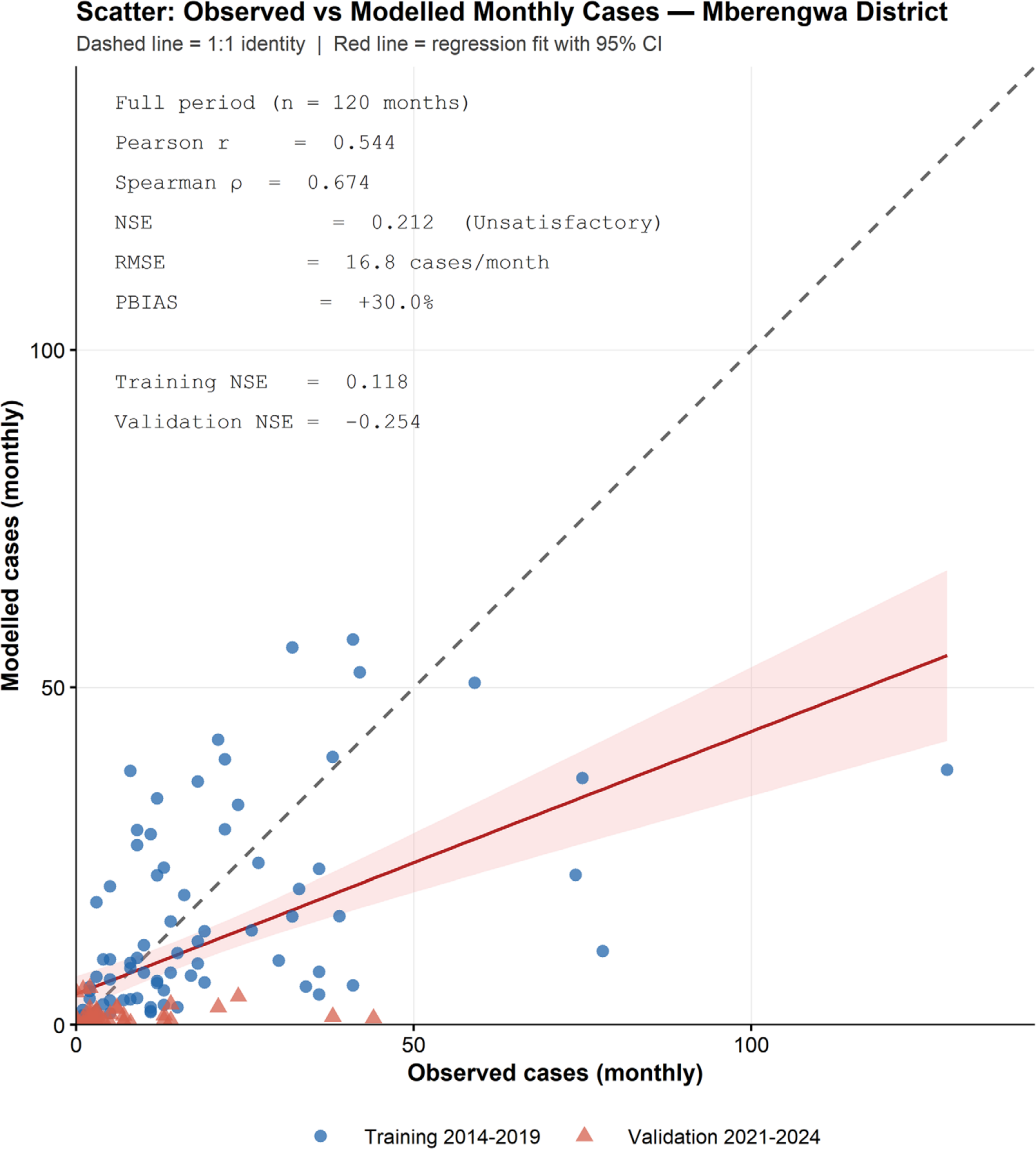
Scatter plot of observed and modelled monthly malaria cases (2014–2024). RMSE, root mean square error.

The SEIR–SEI model was evaluated using 120 months of routine confirmed malaria case data from the Mberengwa District (2014–2024, excluding 2020). Over the full study period, the model achieved a Pearson correlation of *r* = 0.544 and a Spearman's rank correlation of *ρ* = 0.674, with an RMSE of 16.80 cases per month and a PBIAS of +30.0% (Table 4 and Figure 3). The corresponding observed versus modelled scatter plot is presented in Figure 4, and the mean monthly seasonal climatology is shown in Figure S3.

**TABLE 4:** Model validation performance metrics, Mberengwa District SEIR–SEI malaria model.

Metric	Training (2014–2019)	Validation (2021–2024)	Full period (120 months)
**Pearson** r	+0.457	+0.129	+0.544
**Spearman** ρ	+0.541	+0.320	+0.674
**RMSE (cases/month)**	20.06	10.09	16.80
**MAE (cases/month)**	13.04	5.34	9.96
**NSE**	0.118	−0.254	0.212
**PBIAS (%)**	+20.7	+78.6	+30.0

*Note:* 2020 was excluded from all periods due to COVID‐19 surveillance disruption.

Abbreviations: RMSE, root mean square error; MAE, mean absolute error; NSE, Nash–Sutcliffe efficiency; PBIAS, per cent bias.

During the training period (2014–2019), the model recorded Pearson *r* = 0.457, Spearman *ρ* = 0.541, RMSE = 20.06 cases per month, PBIAS = +20.7% and NSE = 0.118. During the independent validation period (2021–2024), the model recorded Pearson *r* = 0.129, Spearman *ρ* = 0.320, RMSE = 10.09 cases per month, PBIAS = +78.6% and NSE = −0.254 (Table 2).

The scaling factor *k* = 4.963 (95% bootstrap CI: 3.850–6.673) indicates a case detection rate of approximately 20.2% (CI: 15.0%–26.0%), meaning that roughly one in five infectious individuals was identified through passive facility‐based surveillance during the study. This aligns with published detection ratios in low‐transmission, pre‐elimination settings in sub‐Saharan Africa, where factors such as partial immunity, asymptomatic cases, and barriers, such as distance to health facilities, limit symptomatic presentation. The systematic overestimation during the validation period (PBIAS = +78.6%) can be mechanistically explained as follows: The model estimates the climate‐driven transmission potential, whereas reported cases reflect the reduced burden after intensified elimination efforts have suppressed transmission below this potential. This divergence is thus evidence of programme success, not model failure.

To evaluate the sensitivity to the 2020 exclusion, three scenarios were compared: one excluding 2020 as in the main analysis (Scenario A); another including the 2020 data with raw reported figures (Scenario B); and a third replacing 2020 data with linear interpolation between 2019 and 2021 values (Scenario C). Incorporating raw 2020 data significantly reduced model performance (*r* = 0.370, NSE = 0.043) compared with the main analysis (*r* = 0.544, NSE = 0.212), indicating that COVID‐19 surveillance disruptions notably affected case reporting in year. The scaling factor *k* showed minimal variation across the scenarios (4.838–5.007). Additionally, all *R*_0_ projections and SSP3‐7.0 climate scenario results remained unchanged across the three scenarios, demonstrating that the exclusion of 2020 does not influence future projections (Table S1).

To show that the mechanistic framework provides additional predictive value beyond a simpler statistical comparison, a seasonal ARIMA model was fitted to the 2014–2019 training data and tested on the 2021–2023 validation dataset. The ARIMA model had a Pearson's *r* of 0.170, NSE of −0.446 and PBIAS of +98.6%. The SEIR–SEI model outperformed ARIMA in terms of Nash–Sutcliffe efficiency (−0.298 vs. −0.446), whereas ARIMA showed nearly double the per cent bias (+98.6% vs. +78.6%), indicating a more systematic overestimation. Both models had poor absolute fit during validation, likely because intensified elimination activities suppressed the observed case counts below the climate‐driven transmission potential. The higher NSE of the SEIR–SEI model highlights the added value of a mechanistic compartmental approach over a purely data‐driven statistical model in the pre‐elimination phase (Figure S4).

### 3.3 Determinants of Transmission and Parameter Uncertainty

Local one‐at‐a‐time (OAT) sensitivity analysis at the baseline (mean $R_{0}$= 2.112, peak $I_{H}$ = 130.2, transmission days ≥ $R_{0}$ 2: 213 days/year) identified the vector mortality quadratic coefficient $q_{2}$ as the parameter with the largest influence on mean annual $R_{0}$, with an NSI of +3.937 (Figure S5). The complementary coefficient $q_{1}$ exerted a strong opposing effect (NSI = −2.380). Human recovery rate $\gamma_{H}$ recorded an NSI of −0.507, and both human infection probability (*b*) and vector infection probability (*c*) each recorded an NSI of +0.504.

**FIGURE 5:**
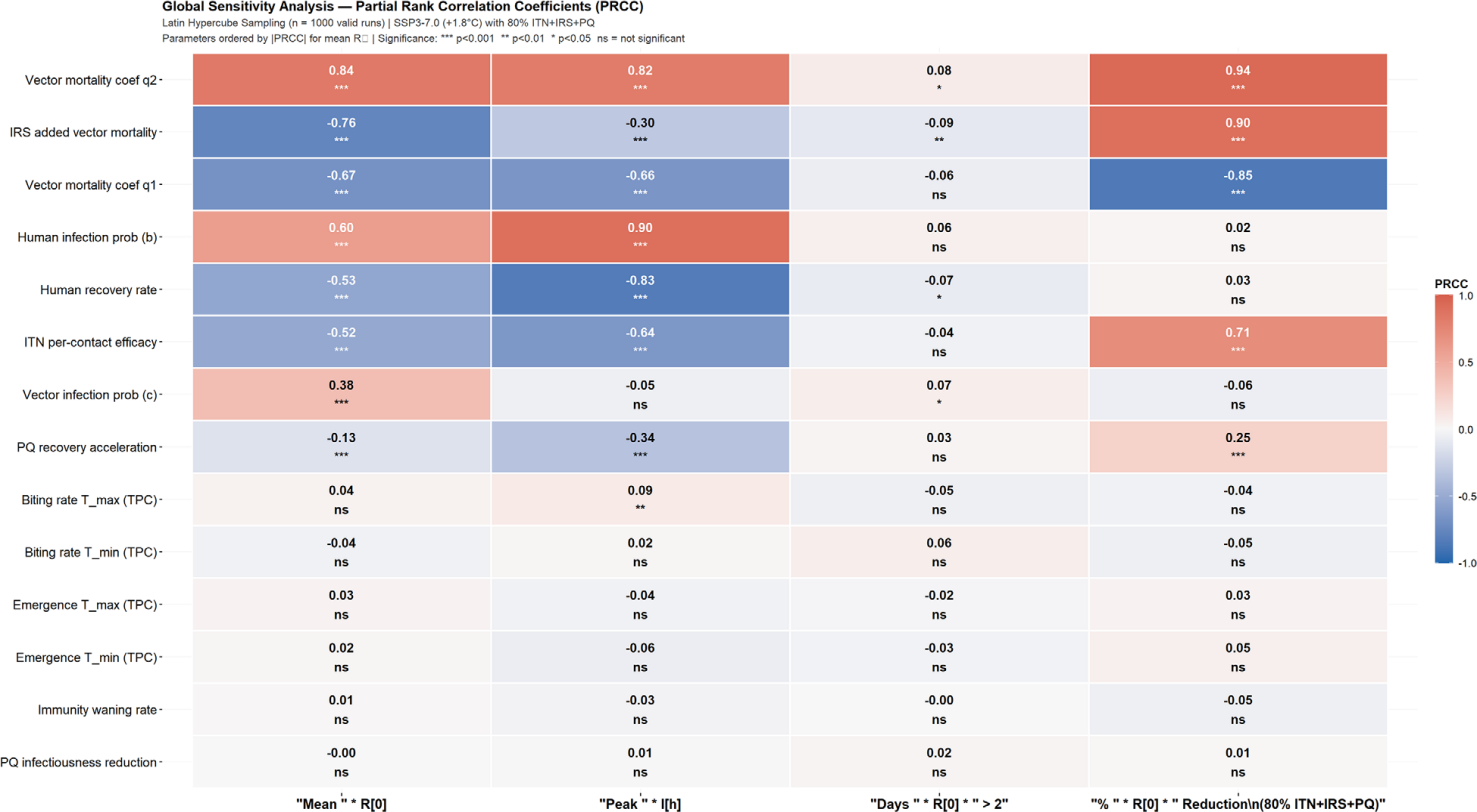
Global sensitivity analysis showing partial rank correlation coefficients (PRCCs) heat map. IRS, indoor residual spraying; ITN, insecticide‐treated net; PQ, primaquine; TPC, thermal performance curve.

Global sensitivity analysis using LHS (*n* = 1000) and PRCCs identified $q_{2}$ as the strongest positive driver of mean annual $R_{0}$ (PRCC = +0.837, *p* < 0.001), followed by IRS‐added vector mortality (PRCC = −0.763, *p* < 0.001) and $q_{1}$ (PRCC = −0.675, *p* < 0.001) as the strongest negative driver. Human infection probability (*b*) (PRCC = +0.598, *p* < 0.001) and human recovery rate $\gamma_{H}$ (PRCC = −0.531, *p* < 0.001) ranked fourth and fifth, respectively (Table 5, Figure 5).

**TABLE 5:** Top five parameters ranked by |PRCC| for mean annual $R_{0}$ in the global sensitivity analysis (*n* = 1000 latin hypercube sampling runs).

Parameter	Category	Mean $R_{0}$	Peak $I_{H}$	Days $R_{0}$ ≥ 2	% $R_{0}$ reduction	NSI
**Vector mortality** $q_{2}$	TPC	+0.837***	+0.830***	+0.112***	+0.940***	+3.937
**IRS added vector mortality**	Intervention	−0.763***	−0.581***	−0.098**	−0.850***	—
**Vector mortality** $q_{1}$	TPC	−0.675***	−0.660***	−0.097**	−0.860***	−2.380
**Human infection prob. (*b*)**	Transmission	+0.598***	+0.900***	+0.074*	+0.021 ns	+0.504
**Human recovery rate (** $\gamma_{H}$)	Transmission	−0.531***	−0.830***	−0.063 ns	−0.018 ns	−0.507

*Note:*, Baseline: mean $R_{0}$ = 2.112, peak $I_{H}$ = 130.2, transmission days ≥ $R_{0}$ 2: 213 days/year.

Abbreviations: ns, not significant; NSI, normalised sensitivity index; TPC, thermal performance curve.

*p < 0.05.

**p < 0.01.

***p < 0.001.

Propagation of parameter uncertainty into seasonal $R_{0}\left(t\right)$ trajectories yielded 95% credible intervals of 0.086–10.61 under baseline conditions, 0.124–11.32 under SSP2‐4.5 (+0.8°C) and 0.167–11.92 under SSP3‐7.0 (+1.8°C), with asymmetric widening of the upper bound during the warm season (Figure S6). The lower credible interval boundary fell below $\;R_{0}\left(t\right)$ = 1 under extreme low‐parameter combinations across all three scenarios.

### 3.4 Projected Transmission Intensification Under Climate Change

**FIGURE 6:**
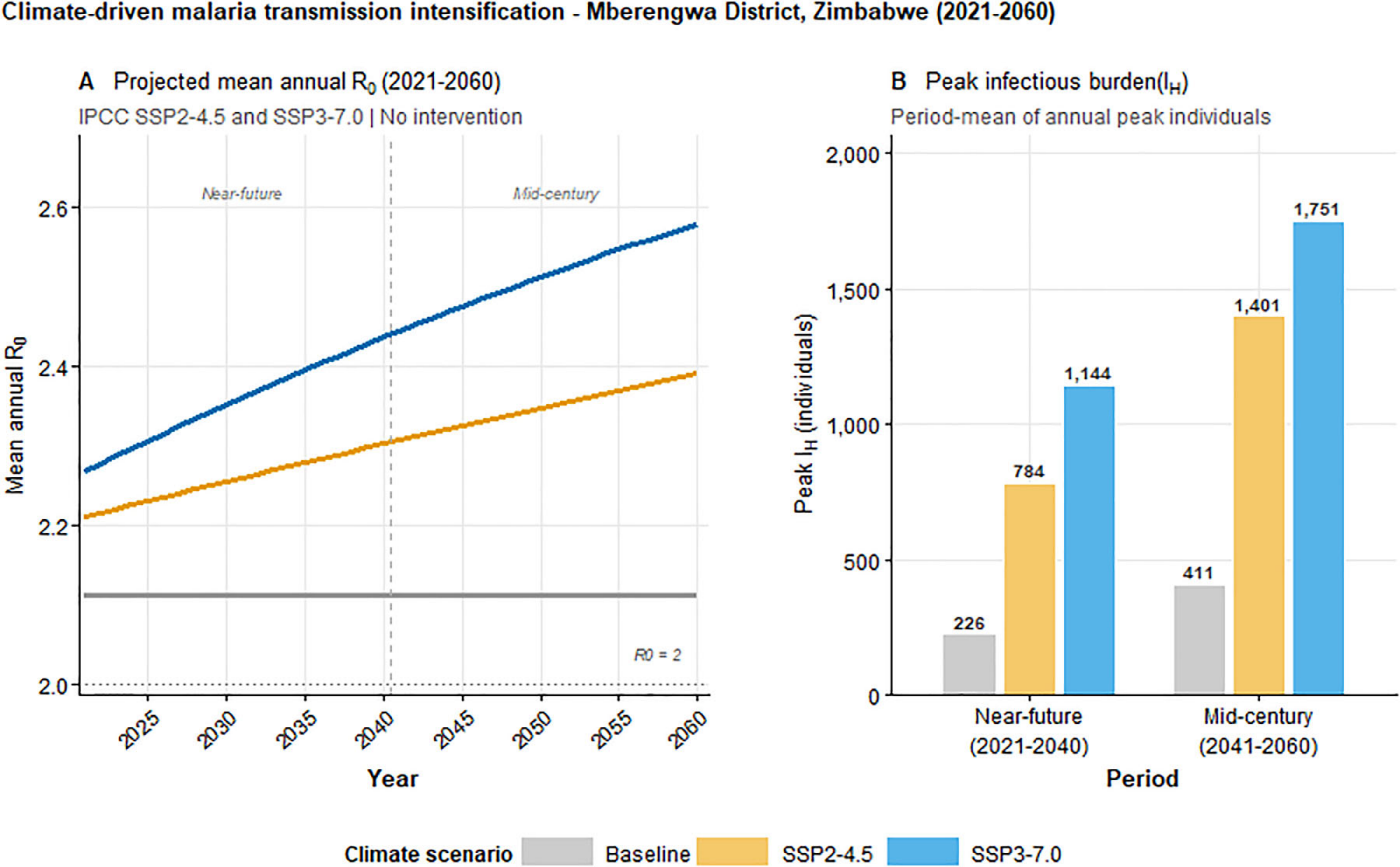
Malaria intensification across climate change scenarios. SSP, shared socioeconomic pathway.(A‐Projected Mean Annual Reproduction Number; B‐Peak Infectious Burden)

Under the SSP2‐4.5 scenario, mean annual $R_{0}$ increased from the baseline of 2.112 to 2.257 in the near‐future (2021–2040) and 2.35 by mid‐century (2041–2060), representing increases of 6.9% and 11.2% above baseline, respectively. Peak infectious prevalence ($I_{H}$) rose from the baseline of 410.6 to 784.5 individuals in the near‐future and 1400 by mid‐century. The high‐intensity transmission season ($R_{0}\left(t\right)$ ≥ 2) extended from 213 days at baseline to 225 days in the near‐future and 234 days by mid‐century (Table 6 and Figure 6).

**TABLE 6:** Projected malaria transmission metrics under baseline and climate change scenarios in Mberengwa District, Zimbabwe, 2021—2060.

Scenario	Period	Mean annual $R_{0}$	Peak $I_{H}$ (individuals)	Days $R_{0}\left(t\right)\geq 2$
**Baseline**	2021–2060	2.112	411	213
**SSP2‐4.5**	Near‐future (2021–2040)	2.257	785	225
	Mid‐century (2041–2060)	2.350	1401	234
**SSP3‐7.0**	Near‐future (2021–2040)	2.354	1144	234
	Mid‐century (2041–2060)	2.514	1751	250

*Note:* Temperature deltas: SSP2‐4.5 near‐future +0.8°C, mid‐century +1.2°C; SSP3‐7.0 near‐future +1.0°C, mid‐century +1.8°C.

Abbreviation: SSP, shared socioeconomic pathway.

Under the SSP3‐7.0 scenario, mean annual $R_{0}$ reached 2.354 in the near‐future (2021–2040) and 2.514 by mid‐century (2041–2060), representing increases of 11.4% and 19.0% above baseline, respectively. Peak $I_{H}$ rose to 1144 individuals in the near‐future and 1750 by mid‐century. The high‐intensity transmission season extended to 234 days in the near‐future and 250 days by mid‐century. $R_{0}\left(t\right)$ remained above 1.0 throughout the entire projection period across all scenarios (Table 4 and Figure S7).

### 3.5 Optimal Integrated Strategies for Elimination Feasibility

All intervention scenarios were examined using detailed dynamic ODE simulations of the model, which captured transient behaviours, feedback loops between intervention coverage and immunity dynamics at the population level, and the delay between coverage changes and reductions in the infectious burden. The basic reproduction number *R*_0_(*T*) functions as the long‐term elimination benchmark (*R*_0_ < 1 under continuous intervention), whereas the simulations identify the peak infectious prevalence (peak $I_{H}$), the months each year with *R*_0_(*T*) ≥ 1, and the progression of infectious burden under different coverage scenarios. Integrating analytical threshold evaluation with full dynamic modelling ensures that intervention assessments consider both the potential for elimination and transmission patterns during each strategy.

**FIGURE 7:**
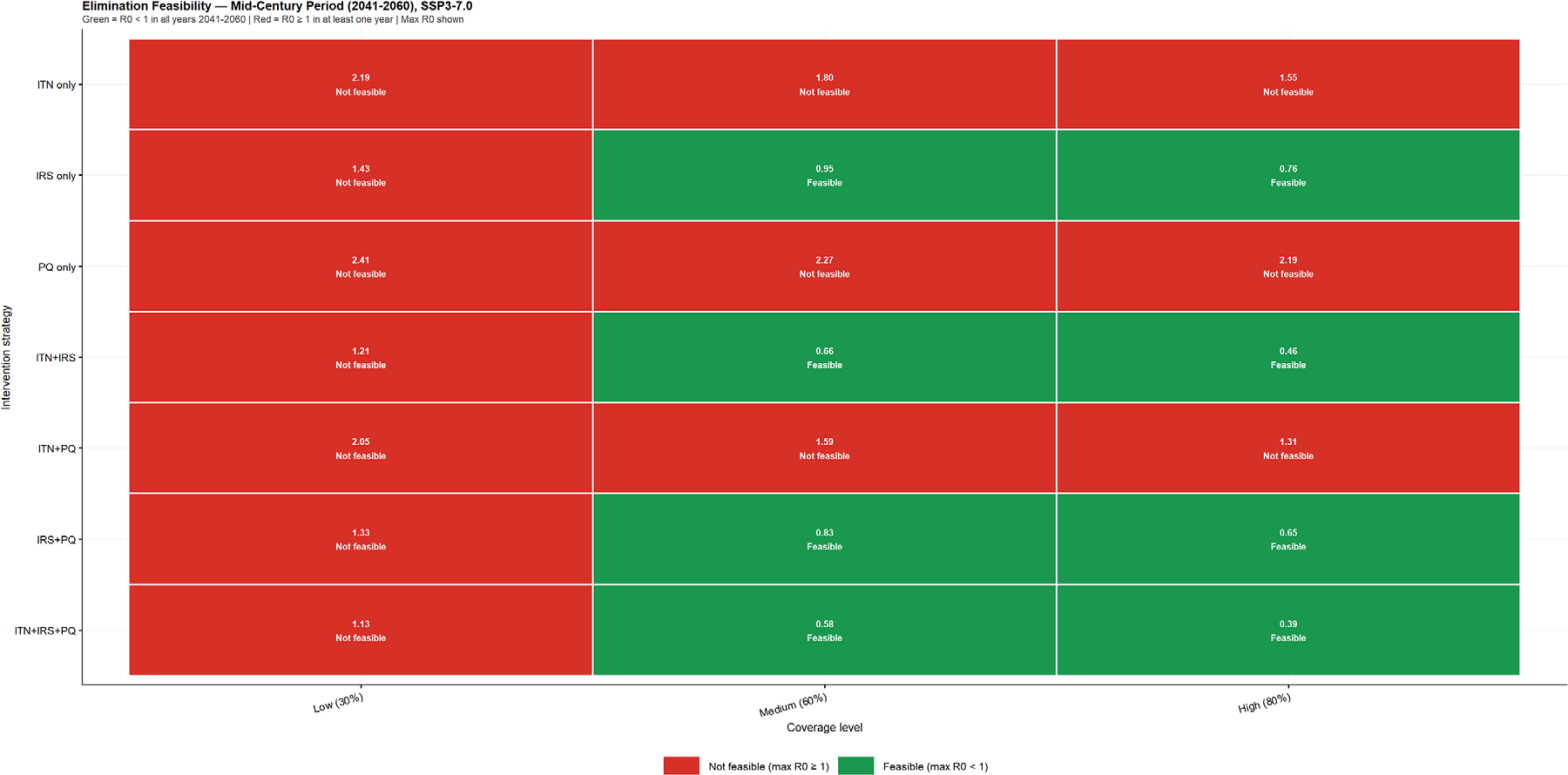
Elimination feasibility heat map for the mid‐century period (2041–2060) under the SSP3‐7.0 high‐emission scenario. SSP, shared socioeconomic pathway.

Under the SSP3‐7.0 mid‐century scenario (2041–2060), only at 80% coverage, the mean annual *R*_0_ was reduced by 70.7%, achieving a maximum *R*_0_ of 0.763 and meeting the elimination feasibility criterion (*R*_0_ < 1.0 in all years). ITN at 80% coverage reduced the mean annual *R*_0_ by 40.0% (max *R*_0_ = 1.546), and PQ monotherapy reduced the mean annual *R*_0_ by 15.2% (max *R*_0_ = 2.186). None of the interventions achieved elimination feasibility individually (Table 7 and Figure 7).

**TABLE 7:** Percentage reduction in mean annual $R_{0}$ and mid‐century elimination feasibility under interventions at 80% coverage.

Intervention (80% coverage)	SSP2‐4.5 red. (mid‐century) (%)	SSP3‐7.0 red. (mid‐century) (%)	Elimination feasibility (max *R*_0_)
**Individual**			
ITN only	38.6	40.0	No (1.55)
IRS only	69.2	70.7	Yes (0.76)
PQ only	14.9	15.2	No (2.19)
**Combination**			
ITN + IRS	80.6	82.4	Yes (0.46)
ITN + PQ	48.1	49.1	No (1.31)
IRS + PQ	73.5	75.2	Yes (0.65)
ITN + IRS + PQ	83.2	85.1	Yes (0.39)

*Note:* Elimination feasibility is defined as maximum annual mean $R_{0}<1$ under the SSP3‐7.0 scenario. Reductions were relative to the climate‐only mid‐century projection for each SSP scenario. All interventions assumed 80% effective coverage applied concurrently.

Abbreviations: IRS, indoor residual spraying; ITN, insecticide‐treated net; PQ, primaquine; SSP, shared socioeconomic pathway.

Among the pairwise combinations at 80% coverage, ITN+IRS reduced the mean annual *R*_0_ by 82.4% (max *R*_0_ = 0.458), and IRS+PQ reduced the mean annual *R*_0_ by 75.2% (max *R*_0_ = 0.647), both achieving elimination feasibility. ITN+PQ reduced the mean annual *R*_0_ by 49.1% (max *R*_0_ = 1.311) and did not achieve elimination. The triple combination of ITN+IRS+PQ at 80% coverage produced the greatest reduction in the mean annual *R*_0_ (85.1%, max *R*_0_ = 0.388) among all strategies tested (Table 7).

At 60% coverage, elimination feasibility was achieved by IRS alone (max *R*_0_ = 0.949), ITN+IRS (max *R*_0_ = 0.664) and IRS + PQ (max *R*_0_ = 0.835), all of which met the *R*_0_ < 1.0 criterion. At 30% coverage, no strategy achieved elimination feasibility under the SSP3‐7.0 mid‐century scenario. The elimination feasibility matrix for all coverage levels and intervention strategies is shown in Figure 7.

### 3.6 Spatial Risk Mapping and Intervention Prioritisation

Spatial analysis of confirmed malaria incidence across the 37 health facility catchments revealed a district mean of 0.8 cases per 1000 population per year (2014–2024, excluding 2020), with substantial heterogeneity across catchments. Global spatial autocorrelation analysis yielded a Moran's *I* of 0.05–0.13 (*p* > 0.05), indicating spatially non‐clustered transmission. The majority of catchments fell into the very low (<1 case per 1000 per year) and low (1–5 cases per 1000 per year) risk categories.

**FIGURE 8:**
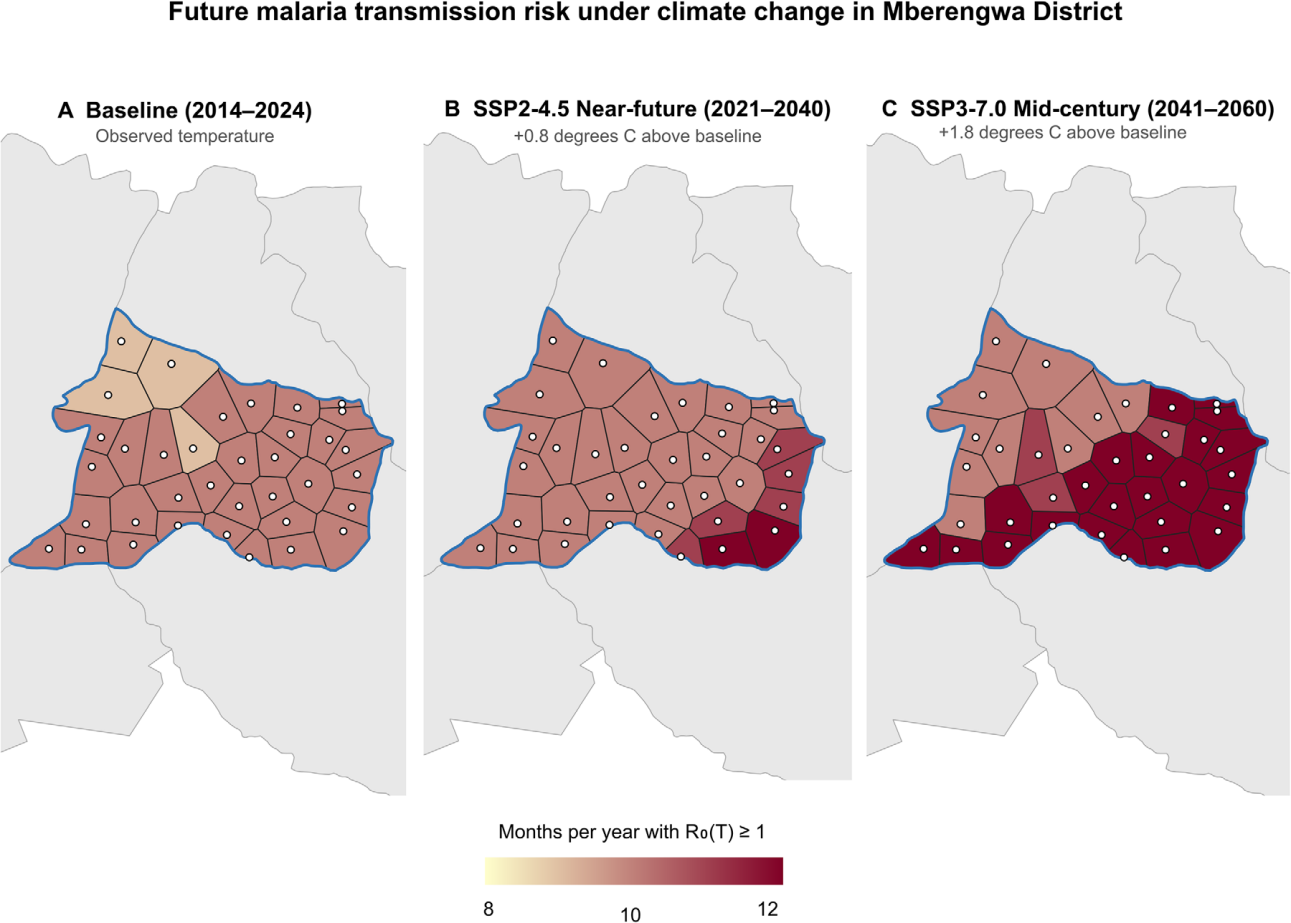
Future malaria transmission risk under climate change in Mberengwa District. (A‐Baseline (2014‐2024); B‐SSP2‐4.5 Near Future (2021‐2040); C‐SSP#‐7.0 Mid‐century (2041‐2060))

The district mean baseline transmission season was 9.9 months per year (defined as months with $R_{0}\left(t\right)$ ≥ 1). Under the SSP2‐4.5 near‐future scenario (2021–2040, +0.8°C), the district mean transmission season extended to 10.2 months. Under the SSP3‐7.0 mid‐century scenario (2041–2060, +1.8°C), the transmission season extended to 11.3 months per year, reaching 12 months per year in the highest risk catchments (Figure 8).

The SRPI classified 5 of 37 catchments (13.5%) as Priority 1, Highest (SRPI ≥ 0.75): Musume (SRPI = 0.94), Mataga RHC (0.76), Gaha (0.76), Masase (0.75) and Sandawana (0.75). A further 19 catchments (51.4%) were classified as Priority 2, High (SRPI 0.50–0.74). In total, 24 of the 37 catchments (64.9%) fell within the two highest priority tiers. The complete SRPI rankings for all 37 catchments are shown in Figure S8.

To assess the robustness of the Priority 1 catchment designations against the equal‐weighting assumption, the SRPI was recalculated using five different weighting schemes: equal weights (W1:1/3 each); incidence‐led (W2:0.50/0.25/0.25); climate‐led (W3:0.25/0.50/0.25); population‐led (W4:0.25/0.25/0.50); and inverse population weighted (W5:0.40/0.40/0.20) (Table S2). Musume was classified as Priority 1 across all five schemes (SRPI_1_ = 0.939 in every case), confirming it as the most consistently high‐priority catchment, regardless of how weights were assigned. Thus, it was a stable, high‐priority catchment. In contrast, Mataga RHC, Gaha and Sandawana were classified as Priority 1 in four of the five schemes, suggesting that they are likely to be Priority 1 as well. Masase and Vurasha were classified as Priority 1 in three of the five schemes, indicating that their classification was sensitive to weight changes. The core group of five Priority 1 catchments, supported by this sensitivity analysis, especially with Musume maintaining its status, includes four other facilities that are consistently ranked as Priority 1 under most plausible assumptions. Facilities initially ranked as Priority 2 or lower did not change to Priority 1 under any scheme, confirming a clear distinction between the top facilities and the rest of the district.

## 4 Discussion

### 4.1 Summary and Interpretation of Key Findings

This study characterised malaria transmission dynamics in the Mberengwa District using a climate‐sensitive SEIR–SEI model integrated with 11 years’ worth of malaria surveillance data and facility‐level spatial risk indices. Model validation with 120 months of surveillance data yielded a Pearson *r* of 0.544 and Spearman ρ of 0.674, demonstrating a moderate to strong ability to capture long‐term seasonal oscillations. The systematic overestimation of clinical cases by the SEIR–SEI model highlights the known discrepancy between the modelled infectious prevalence and passive facility‐based surveillance in low‐endemic areas. The estimated scaling factor *k* = 4.963 (95% CI: 3.850–6.673) suggests a case detection rate of roughly 20%, meaning that for every five infectious individuals in the model, only one visits a health facility and is recorded in DHIS2. The remaining 80% includes asymptomatic or pauci‐symptomatic infections, individuals who avoid care because of distance or cost and cases treated outside the formal health system. This detection rate matches estimates from similar pre‐elimination settings in southern Africa and is supported by the companion study (Paper 2), which found that distance to health facilities was the strongest predictor of severe malaria case presentation in Mberengwa (AOR = 0.06; <5 km vs. >10 km).

The post‐2020 period of increased overestimation (PBIAS = +78.6% in 2021–2024) indicates an additional suppressive influence from intensified elimination efforts, such as IRS, targeted LLIN distribution and active case detection. These interventions reduce the share of the modelled transmission potential that manifests as reported clinical cases. This interpretation aligns with documented disruptions from 2020 to 2022 caused by the COVID‐19 pandemic, which altered health‐seeking behaviours across sub‐Saharan Africa.

The projected 7.8‐fold increase in peak infectious prevalence under SSP3‐7.0 mid‐century conditions arises primarily through the non‐linear amplification of vector biological rates rather than through expansion into currently unsuitable geographical areas. The mean annual *R*_0_ remained above the epidemic threshold throughout the projection period for all scenarios, indicating perennial transmission suitability. These findings are consistent with evidence from temperature‐sensitive modelling studies across sub‐Saharan Africa, demonstrating that marginal temperature increases in the 20–25°C range produce a disproportionate amplification of vectorial capacity in currently endemic settings [3, 7]. The non‐linear response is driven by the TPCs of the vector biting rate and emergence, both of which peak in the 25–30°C range that characterises Mberengwa's projected mid‐century climate [40].

The 86‐day lag between peak *R*_0_(*t*) and peak *I_H_* (*t*) is consistent with the temperature‐dependent extrinsic incubation period of *P. falciparum* and the generation time of the human infectious compartment. Stopard et al. (2021) demonstrated using a mechanistic sporogony model that the extrinsic incubation period at 25°C ranges from approximately 10 to 14 days, with stochastic variation between individual mosquitoes contributing substantially to population‐level transmission delays [41]. Sensitivity analyses identified the vector mortality quadratic coefficients *q*_2_ (NSI = +3.937, PRCC = +0.837) and *q*_1_ (NSI = −2.380, PRCC = −0.675) as the dominant structural drivers of transmission intensity. The primacy of vector mortality in determining malaria *R*_0_ has been well established in the malaria modelling literature. Chitnis et al. [15] identified the mosquito mortality rate as the most sensitive parameter in a comparable SEIR–SEI sensitivity analysis [15]. The dissociation between *R*_0_ and *I_H_* sensitivity, *R*_0_ governed by vector biology while peak I_H_ was dominated by human infection probability (PRCC = +0.900), is consistent with the multi‐scale structure of malaria transmission, where entomological and epidemiological dynamics respond to distinct parameter sets [42].

Model validation against 120 months of surveillance data yielded full‐period Pearson *r* = 0.544, and Spearman *ρ* = 0.674. The positive bias in the validation period (PBIAS = +78.6%, 2021–2024) suggests a systematic overestimation of cases, which may reflect intensified elimination activities not incorporated into climate‐driven baseline equations. The COVID‐19 pandemic caused documented disruptions in malaria health‐seeking behaviour and passive case detection across sub‐Saharan Africa from 2020 to 2022. Heuschen et al. reported significant reductions in outpatient malaria consultations in northern Ghana directly attributable to pandemic‐era movement restrictions, and Duguay et al. demonstrated reduced LLIN usage and health‐seeking behaviour in rural Benin during the same period [43, 44]. Vargas‐Ruiz et al. estimated that COVID‐19‐related disruptions to malaria case management contributed to approximately 5.9 million excess malaria cases across sub‐Saharan Africa in 2020–2021, complicating the interpretation of passive surveillance data from this period [45]. In low‐endemic settings with an API below 5 per 1000, stochastic effects in small case counts are additionally amplified in proportional bias metrics, a recognised limitation of deterministic compartmental models applied to pre‐elimination settings [46].

The non‐significant global spatial autocorrelation (Moran's *I* = 0.05–0.13, *p* > 0.05) across the 37 health facility catchments indicates spatially dispersed transmission foci in the study area. This pattern is consistent with evidence from low‐endemic settings in sub‐Saharan and East Africa, where declining transmission is associated with increasingly focal and spatially heterogeneous distributions of residual cases [47, 48]. The coalescence of residual transmission into discrete foci at low API levels has been documented in southern Africa and is mechanistically consistent with the ecological fragmentation of suitable vector habitats in semi‐arid environments [49]. The SRPI identified five Priority 1 catchments: Musume (SRPI = 0.94), Mataga RHC (0.76), Gaha (0.76), Masase (0.75) and Sandawana (0.75), representing the highest composite burden of current incidence, projected climate exposure and population at risk.

### 4.2 Implications for Policy and Practice

The finding that IRS monotherapy at 80% coverage was the only individual intervention achieving elimination feasibility under SSP3‐7.0 mid‐century conditions (max *R*_0_ = 0.763) is mechanistically consistent with the dominant role of vector mortality in determining transmission intensity, as identified in sensitivity analyses. The superior performance of IRS relative to ITN monotherapy (max *R*_0_ = 1.546) is supported by empirical evidence from Uganda, where discontinuation of IRS was associated with a 5‐fold increase in malaria incidence within 10 months despite universal LLIN coverage and by a systematic review of reactive IRS in pre‐elimination settings that demonstrated a 68% reduction in malaria prevalence (RR = 0.32, 95% CI: 0.13–0.80) [50, 51]. The disparity between ITN and IRS performance in this model has direct operational relevance, given the documented gap between LLIN ownership rates and actual use in the Mberengwa District, which effectively reduces operational coverage below the 80% threshold assumed in the simulation [19]. The triple combination of ITN + IRS + PQ at 80% coverage produced the greatest margin below the elimination threshold (max *R*_0_ = 0.388), and IRS‐containing combinations retained elimination feasibility at 60% coverage across all three tested combinations.

The SRPI framework provides a quantitative basis for sub‐district resource allocation consistent with the WHO Global Malaria Programme's subnational tailoring (SNT) approach, which recommends stratification at the level of health facility catchment areas as countries approach elimination [32]. The concentration of 64.9% of catchments in the two highest priority tiers, combined with the spatially focal transmission pattern, supports a tiered response in which intensive IRS‐based integrated interventions are deployed in the five priority 1 catchments, whereas lower intensity surveillance and maintenance strategies are applied in Priorities 3 and 4 catchments. This approach aligns with the operational principles of the SNT, implemented in over 28 countries from 2022 to 2023, to guide the development of the national malaria strategic plan and Global Fund applications [52]. The projected expansion of the high‐intensity transmission season from 213 days at baseline to 250 days under SSP3‐7.0 mid‐century conditions indicates that the timing of IRS campaigns will require adjustment to accommodate an extended peak transmission window as warming progresses.

The asymmetric widening of the 95% credible interval for *R*_0_(*t*) under SSP3‐7.0 (0.167–11.92) relative to the baseline (0.086–10.61) indicates increasing predictive uncertainty during the warm season under high‐emission scenarios. The 86‐day lag between the *R*_0_(*t*) and *I_H_* (*t*) peaks identified in the seasonal co‐dynamics analysis provides a prospective window for early warning. The integration of model outputs with real‐time meteorological data from the Zimbabwe Meteorological Services Department could operationalise this early warning capacity within existing district health information systems, consistent with the WHO guidance on the transformation of malaria surveillance into a core intervention in pre‐elimination settings [53].

This study provides a locally parameterised, climate‐sensitive transmission model for the Mberengwa District that integrates epidemiological surveillance, vector biology and spatial risk analysis within a unified quantitative framework. The results demonstrate that the projected mid‐century warming under SSP3‐7.0 substantially increases transmission intensity, whereas elimination remains mechanistically feasible through sustained IRS‐based integrated strategies at coverage levels of 60% and above. The SRPI framework identifies five Priority 1 catchments for targeted intervention deployment. Future work should explore hybrid mechanistic‐AI frameworks that combine the biological interpretability of compartmental models with the pattern‐recognition capacity of physics‐informed neural networks, particularly for the post‐elimination transition period where surveillance data become increasingly sparse [16]. These findings provide a quantitative basis for climate‐adaptive malaria elimination planning in the Mberengwa District and a replicable methodological framework applicable to other low‐endemic pre‐elimination settings in Southern Africa.

## 5 Strengths and Limitations of the Study

This study combined a mathematical transmission model, 10 years of health facility‐based surveillance and geographic risk mapping within a single analytical framework. Validating the model against 120 months of confirmed malaria cases from all 37 health facilities strengthened confidence in its ability to replicate seasonal transmission patterns over a clinically meaningful period. The temperature‐response functions were derived from published laboratory studies of *A. arabiensis*, grounding mosquito biology in measured physiology rather than assumed values [5]. Global sensitivity analysis using 1000 LHSs and PRCCs identified the parameters most responsible for model output uncertainty across four transmission metrics simultaneously, providing a more complete picture of structural uncertainty than one‐at‐a‐time approaches [35]. The SRPI stratified all 37 health facility catchments by current malaria burden, projected climate exposure and population at risk, offering a sub‐district prioritisation tool consistent with the WHO guidance on intervention targeting as countries approach elimination [32].

However, this study had several limitations. The model considers temperature as the sole climate driver and excludes other variables, such as rainfall which has an indirect influence through the creation of breeding sites. The model assumes a homogeneous, closed population and does not account for age‐structured immunity, variation in individual exposure or human movement between catchments, factors that may cause the transmission intensity to be underestimated in the highest‐risk foci [15]. Model performance during the 2021–2024 validation period was constrained by a positive per cent bias of 78.6%, reflecting systematic overestimation of case counts. This divergence likely arises because intensified vector control activities and COVID‐19‐related reductions in health facility attendance are not captured by climate‐driven model equations [45]. Temperature forcing was derived from satellite reanalysis at a 25 km resolution fitted to a smooth seasonal curve, which does not resolve the microclimatic variation across Mberengwa's topographically diverse terrain. Although the equal weighting of the three SRPI components has not been formally validated against actual outbreak data, sensitivity tests using five different weighting schemes showed that Musume consistently remained Priority 1. Additionally, Mataga RHC, Gaha and Sandawana were Priority 1 in four of the five schemes. This supports the robustness of core Priority 1 designations.

The deterministic ODE model has limitations in the pre‐elimination context. Because it does not include demographic stochasticity, it cannot simulate random extinction events that become more likely as case numbers approach zero. In pre‐elimination areas where the effective infectious population may be in single digits at the catchment level, stochastic fadeout is a real pathway to elimination that deterministic models miss. Consequently, the model estimates climate‐driven transmission potential rather than providing a probabilistic distribution of outcomes. The development of a stochastic version, such as a continuous‐time Markov chain, would be a valuable direction for future research. Additionally, the assumption of a closed population ignores importation risks from neighbouring districts with higher case rates, such as Mwenezi. Cross‐border movement is a known factor in residual transmission during pre‐elimination; therefore, the model could overestimate elimination chances where border mobility is high. Lastly, the very low case burden during validation, averaging 0.8 cases per 1000 annually, with months recording zero cases, made PBIAS and NSE more sensitive to small prediction errors. Therefore, the validation results should be interpreted cautiously, considering the low transmission setting rather than relying on thresholds designed for higher transmission contexts.

## 6 Conclusions

Climate change is likely to intensify malaria transmission in the Mberengwa District through the non‐linear amplification of vector biological processes, even though the district maintains a pre‐elimination status. Projected warming increases transmission intensity and extends the duration of the high‐risk season. Vector mortality emerges as the dominant control point in this process, indicating that interventions targeting adult mosquito survival, particularly IRS, provide the most effective foundation for climate‐adaptive vector elimination. Malaria elimination is possible under these conditions. Sustained high coverage of integrated vector control can maintain transmission below the epidemic threshold across all warming scenarios. Therefore, the primary constraint is not the availability of effective interventions but the consistency of their implementation. Approaches that rely primarily on reactive or highly targeted spraying during outbreaks may be insufficient in settings where climatic conditions sustain transmission throughout the year, highlighting the importance of maintaining baseline coverage alongside targeted responses. Transmission is spatially concentrated in discrete high‐risk areas rather than being uniformly distributed across the country. A small number of priority catchments account for the highest combined burden of current transmission and future climate exposure, providing a clear basis for targeted intervention. Focusing resources on these areas while maintaining surveillance elsewhere offers a practical and scalable pathway for sustaining malaria elimination under a warming climate in similar settings in the future.

## Author Contributions

Tafadzwa Chivasa led the development of this study as part of the DPhil in Environmental Health degree at the National University of Science and Technology (NUST). Auther Maviza, Mlamuli Dhlamini and Wilfred Njabulo Nunu provided mentorship and reviewed the manuscript. Mlamuli Dhlamini conceptualised the research idea and reviewed and provided substantial input on the draft of this manuscript. The author also provided a visualisation of the study. All authors have read and approved the final version of the manuscript and agreed to its submission for publication. Wilfred Njabulo Nunu is responsible for the overall content as guarantors.

## Funding

The authors have nothing to report.

## Ethics Statement

This study did not require human participation because it focused on analysing existing malaria incidence and climate data obtained from databases. Ethical approval was obtained from the National University of Science and Technology Institutional Review Board on September 3, 2024 (approval number: **NUST/IRB/2024/118**).

## Consent

Informed consent was not necessary for this study because only longitudinal secondary data were used, and there was no direct interaction with individual participants. The findings will be disseminated to relevant stakeholders through peer‐reviewed journals, meetings, conferences and seminars.

## Conflicts of Interest

The authors declare no conflicts of interest.

## Supporting information




**Figure S1**: Profile Likelihood for Scaling Factor *k*.

**Figure S2**: Seasonal co‐variation of $R_{0}$ and the infectious human population $\;I_{H}$.

**Figure S3**: Mean monthly seasonal climatology of observed and modelled malaria cases (2014–2024).

**Figure S4**: Out‐of‐sample validation performance of the SEIR–SEI model compared with a seasonal ARIMA benchmark.

**Figure S5**: Local sensitivity analysis (one‐at‐a‐time) bar chart.

**Figure S6**: Parameter uncertainty in seasonal $\;R_{0}\left(t\right)$, with 95% and 50% credible intervals across climate scenarios.

**Figure S7**: Projected mean basic reproduction number (*R*_0_) under climate change scenarios in the absence of intervention.

**Figure S8**: Intervention priority ranking for health facility catchments in the Mberengwa District.

## Data Availability

The datasets generated and analysed in the current study are available from the corresponding author upon reasonable request.
